# Knock down of TIMP-2 by siRNA and CRISPR/Cas9 mediates diverse cellular reprogramming of metastasis and chemosensitivity in ovarian cancer

**DOI:** 10.1186/s12935-022-02838-x

**Published:** 2022-12-30

**Authors:** Ruth M. Escalona, Simon Chu, Elif Kadife, Jason K. Kelly, George Kannourakis, Jock K. Findlay, Nuzhat Ahmed

**Affiliations:** 1 Department of Obstetrics and Gynaecology, University of Melbourne, Melbourne, VIC 3052 Australia; 2 Centre for Reproductive Health, Hudson Institute of Medical Research and Department of Translational Medicine, Monash University, Clayton, VIC 3168 Australia; 3 Fiona Elsey Cancer Research Institute, Suites 23, 106-110 Lydiard Street South, Ballarat Technology Park Central, Ballarat, VIC 3350 Australia; 4 Centre for Endocrinology and Metabolism, Hudson Institute of Medical Research and Department of Translational Medicine, Monash University, Clayton, VIC 3168 Australia; 5 School of Science, Psychology and Sport, Federation University, Mt Helen, VIC 3350 Australia

**Keywords:** Ovarian cancer, TIMP-2, siRNA, CRISPR/Cas-9, MMP2, MMP14, Proliferation, Migration, Invasion

## Abstract

**Background:**

The endogenous tissue inhibitor of metalloproteinase-2 (TIMP-2), through its homeostatic action on certain metalloproteinases, plays a vital role in remodelling extracellular matrix (ECM) to facilitate cancer progression. This study investigated the role of TIMP-2 in an ovarian cancer cell line in which the expression of TIMP-2 was reduced by either siRNA or CRISPR/Cas9.

**Methods:**

OVCAR5 cells were transiently and stably transfected with either single or pooled TIMP-2 siRNAs (T2-KD cells) or by CRISPR/Cas9 under the influence of two distinct guide RNAs (gRNA1 and gRNA2 cell lines). The expression of different genes was analysed at the mRNA level by quantitative real time PCR (qRT-PCR) and at the protein level by immunofluorescence (IF) and western blot. Proliferation of cells was investigated by 5-Ethynyl-2′-deoxyuridine (EdU) assay or staining with Ki67. Cell migration/invasion was determined by xCELLigence. Cell growth in vitro was determined by 3D spheroid cultures and in vivo by a mouse xenograft model.

**Results:**

Approximately 70–90% knock down of TIMP-2 expression were confirmed in T2-KD, gRNA1 and gRNA2 OVCAR5 ovarian cancer cells at the protein level. T2-KD, gRNA1 and gRNA2 cells exhibited a significant downregulation of MMP-2 expression, but concurrently a significant upregulation in the expression of membrane bound MMP-14 compared to control and parental cells. Enhanced proliferation and invasion were exhibited in all TIMP-2 knocked down cells but differences in sensitivity to paclitaxel (PTX) treatment were observed, with T2-KD cells and gRNA2 cell line being sensitive, while the gRNA1 cell line was resistant to PTX treatment. In addition, significant differences in the growth of gRNA1 and gRNA2 cell lines were observed in in vitro 3D cultures as well as in an in vivo mouse xenograft model.

**Conclusions:**

Our results suggest that the inhibition of TIMP-2 by siRNA and CRISPR/Cas-9 modulate the expression of MMP-2 and MMP-14 and reprogram ovarian cancer cells to facilitate proliferation and invasion. Distinct disparities in in vitro chemosensitivity and growth in 3D culture, and differences in tumour burden and invasion to proximal organs in a mouse model imply that selective suppression of TIMP-2 expression by siRNA or CRISPR/Cas-9 alters important aspects of metastasis and chemosensitivity in ovarian cancer.

**Supplementary Information:**

The online version contains supplementary material available at 10.1186/s12935-022-02838-x.

## Background

Ovarian cancer, commonly known as a ‘silent killer’ because of its asymptomatic nature of disease progression, is the most lethal of all gynaecological cancers. In 2021, it was estimated that 1720 women were newly diagnosed with ovarian cancer in Australia, and approximately 1100 died of this disease, making the mortality rate approximately 5% of all female deaths from cancer (https://www.canceraustralia.gov.au/cancer-types/ovarian-cancer/statistics). Most ovarian cancer patients are diagnosed at an advanced stage, with extensive peritoneal metastasis, making the 5-year survival rate as low as 30% [1]. Unlike other cancers, ovarian carcinomas rarely metastasize beyond the peritoneal cavity [2, 3]. In addition, the presence of multicellular aggregates or spheroids in patients’ ascites is a contributing factor for metastasis and chemoresistance. Spheroids contain ovarian cancer stem cells (CSCs) with characteristics for self-renewal, ability to produce differentiated progenies, higher migratory/invasive potential, changed metabolism and augmented chemoresistance [4, 5].

Ovarian cancer patients diagnosed with advanced stage disease routinely undergo cytoreductive surgery usually followed by six cycles of chemotherapy (commonly taxane and platinum-based chemotherapies) [6]. This initial treatment is successful in 80% of cases, and most patients then undergo a short period of remission (few months). However, relapse is inevitable in almost all patients leading to further cycles of chemotherapy. These consecutive episodes of relapse followed by different lines of chemotherapy treatment continues until patients become refractory to chemotherapy and succumb to death [7]. Peritoneal metastasis and chemoresistance-associated relapse are major prognostic factors for poor survival in ovarian cancer patients. Therefore, it is important to understand the factors in the tumour microenvironment (TME) which may be contributing to the underlying molecular mechanisms of peritoneal metastasis and chemoresistance, which if specifically targeted may reduce the mortality rates in these patients.

The expression and activities of metzincins [matrix metalloproteinases (MMPs) and disintegrin and metalloproteinases (ADAMs)] are aberrantly expressed during most pathologic conditions, including cancer [8, 9]. In that scenario, enhanced expression of MMPs affects the remodelling of the extracellular matrix (ECM) required for cancer progression and relapse; these molecules are also involved with the inhibition of apoptosis, remodelling of tumour vasculature and cellular differentiation required by the rapidly evolving progressive tumours [8, 9]. The enzymatic activity of metzincin proteinases is controlled to a certain extent by the endogenous expression of a family of 4 human tissue inhibitors of metalloproteinases (TIMPs). The 4 TIMPs (TIMP-1, TIMP-2, TIMP-3, and TIMP-4) can inhibit the 23 human MMPs with varying degrees of inhibition depending on the makeup of specific TME [8]. However, MMP independent functions for TIMPs have also been reported [8, 10]. These may occur through the activation of a range of signalling pathways which include mitogen activated protein kinase (MAPK), cyclic adenosine monophosphate (cAMP)-protein kinase A, Src kinases and activation of Ras pathways to facilitate cell growth for cancer progression [11–15]. Our previous work showed that TIMP-2 and TIMP-3 proteins are highly expressed in ovarian carcinomas compared to normal ovarian tissues and benign tumours; TIMP-1 and TIMP-2 proteins are excessively secreted in ascites of ovarian cancer patients; and the mRNA expression of TIMP-2 is significantly lower in ascites-derived tumour cells isolated from chemotherapy treated recurrent patients compared to chemo naive patients [16]. These findings are consistent with TIMPs being multifunctional proteins having varied roles in ovarian cancer progression and chemoresistance.

A widely used method for understanding gene function at the cellular level is to reduce or completely disrupt its normal expression using RNAi technology [17]. However, RNAi results in transient (siRNA) or stable (shRNA) phenotypic changes in cells resulting from partial loss of gene expression and function through transcriptional regulation [18]. The CRISPR/Cas9 technology on the other hand induces gene editing by slicing the DNA at a region of interest and then letting the endogenous DNA repair processes heal the cut regions [19, 20]. The outcome can result in either a complete ‘knock out’ or ‘knock down’ (suppression) of gene expression/function [21–23].

In this study we created transient and stable TIMP-2 knocked down transfectants in the OVCAR5 cell line by both the siRNA and CRISPR/Cas9 methods, respectively, and systematically studied how a reduction in TIMP-2 expression affects proliferation, migration/invasion, chemosensitivity, spheroid formation (capacity of cells to form cell aggregates in a floating condition), and tumour progression and overall survival in a mouse xenotransplantation model. We demonstrate that suppression of TIMP-2 by both methods can have different functional outcomes in the resultant cell lines in relation to chemosensitivity in vitro, and growth and tumorigenic potential in in vivo.

## Methods

### Cell culture

Human OVCAR5 ovarian cancer cell line was obtained from Professor David Bowtell (Peter MacCallum Cancer Centre, Parkville, Australia). The cells were maintained in RPMI-1640 (Sigma-Aldrich, Sydney, Australia); supplemented with l-glutamine (2 mM), and antibiotics (Fungizone, Streptomycin and penicillin 1% v/v) and FBS (10% v/v). Cell lines were maintained at 37 °C in 5% CO_2_. Cell lines were passaged twice a week as they reached a confluence of 65–80%.

### Transient transfection of OVCAR5 cells with TIMP-2 specific siRNA

siRNA transfection in the OVCAR5 cell line was performed as described previously [24]. In brief, three small interfering RNA (27mer, siRNA A, B, C) duplexes against human TIMP-2 (OriGene Technologies, SR304838, MD, USA) and a pooled siRNA (A + B + C) at a final concentration of 3 nM were used to knock down the expression of TIMP-2 (T2-KD) in OVCAR5 cell line. A Universal non-targeting siRNA duplex was used as a Control (Cont) (OriGene Technologies, SR30004, MD, USA). Parental cells were treated with Lipofectamine 2000 (Invitrogen, Thermo-Fisher Scientific, CA, USA) transfection reagent only (no siRNA). As described previously, off-target effects were reduced by optimizing the lowest concentration of siRNA needed (using a range of 1 to 10 nM), and transfection efficiency was optimized using 15 nM siGLO™ Red Transfection Indicator (Dharmacon) as per manufacturer’s instructions [24].

### CRISPR/Cas-9 transfection of OVCAR5 cells

The OVCAR5 cell line was co-transfected with a gRNA plasmid and donor fragment [TIMP-2 Human Gene Knockout Kit (CRISPR), KN209796, Origene Rockville, Maryland, US]. Two gRNA plasmids against the TIMP-2 sequence were used: KN209796G1, TIMP-2 gRNA vector 1 in pCas-Guide vector, 3–5 µg, Target Sequence: AGCAGCTGCAGGCGTCGGCC (called gRNA1); and KN209796G2, TIMP-2 gRNA vector 2 in pCas-Guide vector, 3–5 µg, Target Sequence: CGCACCCTGCGGCTGGCGCT (called gRNA2). The linear donor fragment, KN409796D, contained a green fluorescent protein (GFP) and puromycin (LoxP-EF1A-tGFP-P2A-Puro-LoxP). One gRNA/Cas-9 vector and donor fragment (GFP and puromycin) plus Lipofectamine 2000 at a 1:3 ratio was used to transfect the OVCAR5 cells. Forty-eight hours after transfection (passage 1), the cells were split 1:6 and grown for a further 3 days. A puromycin “death” curve for the OVCAR5 cell line was established using an MTT assay (see below). Maximum cell death occurred at a puromycin concentration of 3 µg/mL or higher. Hence, a 3 µg/mL concentration was used for selecting the TIMP-2 edited CRISPR/Cas9 cells and these cells were considered as puromycin resistant. Cells were then passaged every 4 days until passage 10, when the cells were seeded into flasks containing complete growth medium plus puromycin (3 µg/mL). Puromycin resistant TIMP-2 edited CRISPR/Cas9 cells were then GFP sorted by Flow cytometry using the BD FACS Aria (BD, NSW, Australia). A GFP positive area was selected, and cells were then collected and placed in 25cm^2^ flasks with complete medium plus 3 µg/mL of puromycin and incubated at 37 °C in 5% CO_2_ humidity.

### Immunofluorescence (IF)

This technique was performed on OVCAR5 control, and siRNA, and CRISPR/Cas-9 transfected cell lines as described previously [24]. Briefly, 1 × 10^4^ cells in 8-well chamber slides were fixed with paraformaldehyde (PFA)/PBS solution, permeabilized by 0.1 (v/v) Triton X-100 (Sigma-Aldrich) in PBS, washed in cold PBS, incubated with blocking buffer (1% BSA/PBS) for 2 h and then treated with primary antibody overnight at 4 °C. Primary antibodies used were MMP-14 (MT1-MMP, Cell Signaling, Massachusetts, US), MMP-2 (R&D Systems, Melbourne, Australia); TIMP-2 (TIMP-2 (Origene; Rockville, US); and ki67 (anti-ki67 antibody [SP6], Abcam, Cambridge, UK). This was followed by staining the cells with appropriate secondary antibodies (1:200 dilutions) in blocking buffer for 2 h. Nuclei were stained with DAPI (4′,6-diamidino-2-phenylindole) (Invitrogen, Carlsbad, USA) at a 1:2000 dilution for 10 min at room temperature. Fluorescence imaging was observed using an OLYMPUS BX53F upright microscope (Olympus, Tokyo, Japan). Imaging and the intensity of fluorescence was measured according to DAPI location using FIJI analysis software [ImageJ software 1.51j8 (Wayne Rasband National Institute of Health, USA)]. Acquisition of equal intensity was set up before each experiment to avoid biased measurements. This was repeated 4–9 times for each photograph and two images were taken for each well. The fluorescence intensity of each cell group was then graphed, giving a mean average intensity ± SEM.

### Western blot (WB)

Western blot was performed on cell lysates using SDS-PAGE by the methods described previously [25]. Total protein (40 µg) was separated by SDS-PAGE gel (10% resolving; Bio-Rad Laboratories) and transferred to a PDVF membrane. After blocking of non-specific binding with 5% BSA in Tris-buffered saline for 30 min, the membranes were treated with primary antibodies [anti-TIMP-2 (1:460; Abcam, Cambridge, UK) or anti-GAPDH (1:500; Novus Biologicals, Colorado, USA) at 4 °C overnight followed by treatment with secondary horseradish peroxidase-conjugated anti-mouse IgG (1:2000; DAKO, Agilent Technologies, CA, USA) for 1 h at room temperature. Protein bands were visualized using the enhanced chemiluminescence reagents (Bio-Rad Laboratories, Melbourne, Australia). Quantification of densitometry of separated bands was performed with Image Lab software version 6.0.0 (Bio-Rad Laboratories, Melbourne, Australia).

### TIMP-2 ELISA assay

ELISA (enzyme-linked immunosorbent assay) was performed using the MILLIPLEX® MAP Human TIMP Magnetic Bead Panel 2 (TIMP-1, -2 and -3), as per manufacturer’s instructions (EMD Milipore Corporation, USA) and as described previously [16]. Briefly, 30 µg cell conditioned media were added to a 96-well microplate in triplicate. After removal of Wash buffer treatment, then 50 µL of assay buffer (containing 30 µg of sample) was added, plus 25 µL of TIM-2 beads and plates were incubated overnight at 4 °C. Next day, the microplate was washed twice using a solid plate handheld magnet (EMD Millipore) and 200 µL of wash buffer, followed by 25 µL of detection antibodies and incubated at room temperature for 1 h. This was followed by 25 µL of Streptavidin–phycoerythrin and incubated for another 30 min at room temperature and washed again twice more. Finally, 100 µL of Bio-Pex® Sheath fluid (Bio-Rad, Austin, Texas, USA) was added to all the wells and the plate was then run on a Luminex 200TM (Bio-Rad, Austin, Texas, USA) equipped with BioPlex Manager 5.0 software (Bio-Rad, Austin, Texas, USA). The minimum detectable concentration for TIMP-2 was 18.4 pg/mL for 2 h incubation. The concentration of TIMP-2 protein was then determined taking into consideration any dilution factor used for each sample and then divided by the total µg used per sample.

### Proliferation assays

#### MTT assay

This was performed as described previously [16, 24]. Control, siRNA, and CRISPR/Cas9 TIMP-2 knocked down OVCAR5 cells were either left untreated or treated with paclitaxel (PTX) at varying concentrations (0 to 320 µg/mL) for 48 h. For puromycin cell death, OVCAR5 cells were incubated for 48 h in different puromycin concentrations ranging from 0 nM to 320 µg/mL. The culture medium was replaced with 100 µL of 3-(4,5-dimethylthiazol-2-yl)-2,5-diphenyltetrazolium bromide) (MTT) solution (Sigma-Aldrich) dissolved in 1× PBS solution (0.5 mg/mL, final concentration) (Sigma-Aldrich). After 2 h incubation, the MTT solution was replaced with 100 µL of dimethyl sulfoxide (DMSO). Absorbance was read at OD595nm using the CLARIOstar Plate Reader (BMG Labtech, Germany) and analysed by MARS Data Analysis Computer Software (BMG Labtech, Mornington, Victoria, Australia). Each concentration was repeated in quadruplicates and each experiment was repeated 3 times.

#### EdU assay

This assay was performed using the Click-ITTM Plus EdU Flow Cytometry Assay Kit (Invitrogen/Thermo Fisher Scientific North Ryde, NSW, Australia) as described previously [24]. Briefly, 2 × 10^4^ control and transfected cells were treated with 5-Ethynyl-2′-deoxyuridine (EdU) at a final concentration of 10 µM for 1 h at 37 °C. Cells were then trypsinized, fixed in Click-IT fixative followed by staining with Alexa Fluor 647 Picoyl Azide and 20 µg/mL propidium iodide. Fixed and stained cells were analysed by flow cytometry using 633/635 nm excitation with a red emission filter for the detection of Alexa Fluor 647 Azide. Cells for which EdU or Alexa 647 Azide were omitted were used as negative controls for EdU staining. Experiments were performed in quadruplicates and each experiment was repeated 2 times.

### Migration/invasion assays

These assays were performed using the Roche xCELLigence DP instrument as described previously [24]. Briefly, for the migration assays, a 16-well CIM plate (Roche) was equilibrated with pre-warmed serum free media (Gibco® Opti-MEM™ Media (Thermo-Fisher Scientific, Australia). Upper and lower chambers of the CIM plate were filled with 160 µL of complete serum medium and placed in 37 °C incubator for 1 h to allow the plate to equilibrate. Approximately, 40,000 cells suspended in 130 µL Gibco® Opti-MEM™ Media (Thermo-Fisher Scientific, Australia) were seeded on the top compartment of the pre-equilibrated 16-well CIM plate (Roche). Readings were taken every 15 min for ~ 80 h. Each plate contained two duplicate wells and each experiment was repeated 3 times, the mean results were illustrated graphically using PRISM software.

For invasion assays, the CIM plates were coated with 20 µL of Matrigel. Cells (4 × 10^4^) suspended in 130 µL Gibco® Opti-MEM™ Media (Thermo-Fisher Scientific, NSW, Australia) were seeded on the top compartment of the pre-equilibrated 16-well CIM plate (Roche). Readings were taken every 15 min for ~ 40 h. Each plate contained two duplicate wells and each experiment was repeated 3 times. Linear regression analysis of two slopes arising from Cont and T2-KD cells were used to assess significance.

### RNA extraction, quantitative and relative real-time PCR (qRT-PCR)

RNA was extracted from RNAi-treated Cont, T2-KD, CRISPR/Cas9 TIMP-2 edited gRNA1, gRNA2, CRISPR control and parental OVCAR5 cells using TRIzol® reagent (Ambion-Life Technologies, Carlsbad, CA, USA) followed by the chloroform: phenol method as described previously [16]. Five hundred ng of total RNA was reverse transcribed using the high-capacity cDNA Reverse Transcription Kit (Applied Biosystems, CA, USA) and qRT-PCR amplification was performed using the Applied Biosystems ViiA 7 Real-Time PCR (Thermo Fisher Scientific, NSW, Australia) as described previously [16, 26]. Additional file 1: Table S1 lists the sequences and accession numbers of genes analysed. Data are presented as relative expression normalized to housekeeping gene 18S. The experiments were repeated three times in triplicate.

### 3D-Spheroid cultures

The CRISPR/Cas9 transfected OVCAR5 ovarian cancer cells, gRNA1 and gRNA2 and their respective controls, were seeded at a concentration of 1 × 10^5^ cells per well on Ultra- low-attachment 6 well culture plates (Costar, New York, USA). Cells were grown at 37 °C in 5% CO_2_ humidity for 2, 4 and 8 days. Additional growth medium was added at day 4 for cells grown to 8 days. Cells seeded onto normal 6-well culture plates and grown as a monolayer were collected the next day and labelled as day 0. At the end of culture, cell spheres and monolayers were collected and prepared for RNA and Western blot as described above. The sphere forming ability of cells was photographed over 8 days using a Motic AE31 Elite Inverted Phase Contrast Microscope and Motic Image Plus 2.0 software (Motic China Group Co. Ltd). Images were acquired using a 4× or 10× objectives.

#### Spheroid attachment

Cells grown as floating multicellular aggregates (spheroids) were allowed to grow for 10 days after which they were transferred by careful pipetting (1 mL tips were used) onto either an 8-well-chamber slide or 6-well culture plates with fresh complete media. Cells transferred onto 6-well culture plates were imaged either immediately (0 h), or at 2, 10 or 24 h after transfer using the Motic AE31 Elite Inverted Phase Contrast Microscope and Motic Image Plus 2.0 software (Motic China Group Co. Ltd). Images were obtained using a 4× or 10× objective. Cells transferred onto 8-well-chamber slides were allowed to grow for 24 h before fixing, permeabilizing and staining as described in “Immunofluorescence (IF)” section.

### Animal studies

Animal ethics statement: This study was carried according to the recommendations in the Guide for the Care and Use of the Laboratory Animals of the National Health and Medical Research Council of Australia. The experimental protocol was approved by the Animal Ethics Committee of University of Melbourne (Project #1814509).

#### Intraperitoneal injection of ovarian cancer cell lines

Female Balb/c nu/nu mice (6–8 weeks old) were obtained from the Animal Resources Centre, Western Australia. Cells (5 × 10^6^) OVCAR5 control or CRISPR/Cas-9 edited (gRNA1 and gRNA2) were injected intraperitoneally (IP) into each mouse (n = 5/group). Mice well-being were monitored daily, and mice were euthanized once humane endpoints were observed in accordance with the ethics approval. This included tumour burden hampering mobility of mice, body weight loss greater than 15% of initial body weight, and any signs of distress including abnormalities in motility and respiration.

#### Haematoxylin and eosin (H&E) staining and immunohistochemical analyses of xenografts

Mouse organs and tumours were processed and stained by staff at the Anatomical Pathology Laboratory Services, the Royal Children’s Hospital, Melbourne, Australia or at Monash Histology Department, Monash University, Australia as described previously [25]. The formalin-fixed tissues were dehydrated with ethanol (70% for 2 h, 90% for 1 h), followed by immersion in xylene for 2 h and paraffin embedding. Tissue sections were cut at 4 μm thickness.

For H&E staining, sections were deparaffinised, rehydrated, and stained for 3 min with haematoxylin (Australian Biostain Pty Ltd, Traralgon, VIC, Australia). A rinse with 0.25% acid alcohol and Scott’s tap water substitute was followed by staining with eosin (Amber Scientific, Midvale, WA, Australia) for 2 min. A final rinse with absolute alcohol and xylene was performed prior to mounting.

Immunohistochemical staining for mouse tissues was performed with a Ventana Benchmark Immunostainer (Ventana Medical Systems, Inc. Tucson, AZ, USA) as described previously [25]. Briefly, the tumour sections were de-waxed using Ventana Ez Prep and endogenous peroxidase quenched with Ventana Universal DAB inhibitor. Slides that were stained for mouse monoclonal primary antibodies were blocked for mouse-on mouse reactions with AffiniPure Fab Fragment Goat Anti-Mouse IgG (H+L) (Jackson ImmunoResearch Labs Inc, PA, USA) at a concentration of 200 µg/mL diluted in antibody diluent (DAKO, CA, USA) for one hour at room temperature. However, slides stained for rabbit polyclonal primary antibodies were blocked with Serum Free Protein Block (DAKO, CA, USA) for 30 min at room temperature. All slides were then washed in 1× Envision Flex Wash Buffer (DAKO, CA, USA) for 5 min at room temperature. Slides were then incubated with the appropriate diluted primary antibodies for 1 h at room temperature [(all primary antibodies were diluted in antibody diluent (DAKO, CA, USA)]. This was followed with 2 × 5 min buffer washes at room temperature (in 1× Envision Flex Wash Buffer (DAKO, CA, USA). The sections were counter-stained with Ventana Haematoxylin and Blueing solution. Primary antibody staining was visualized using ultra-View Universal DAB detection Kit (Roche, Basel, Switzerland). All images were captured on an Evos FL Auto 2 microscope. Stained immunohistochemical slides were scanned at 40× magnification using the Aperio Scanscope XT (Aperio-Leica Microsystems Pty Ltd).

### Statistical analysis

Unpaired Mann–Whitney’s t-test was used when only two treatment groups were compared. However, a One-Way ANOVA was used for comparison between more than two treatment groups. Data are presented as mean ± standard error of the mean (SEM). xCELLigence data was analysed by linear regression analysis and presented as the standard deviation (SD) of the mean. For statistical significance, the probability levels adopted were p < 0.05(*), p < 0.01(**), p < 0.001 (***) and p < 0.0001 (****). All data were analysed by Graph Pad PRISM software and Microsoft Excel 2016. All experiments were performed for a minimum of three times (unless otherwise indicated) in triplicate.

## Results

### TIMP-2 knockdown in the OVCAR5 ovarian cancer cell line using siRNA technology

**Fig. 1:**
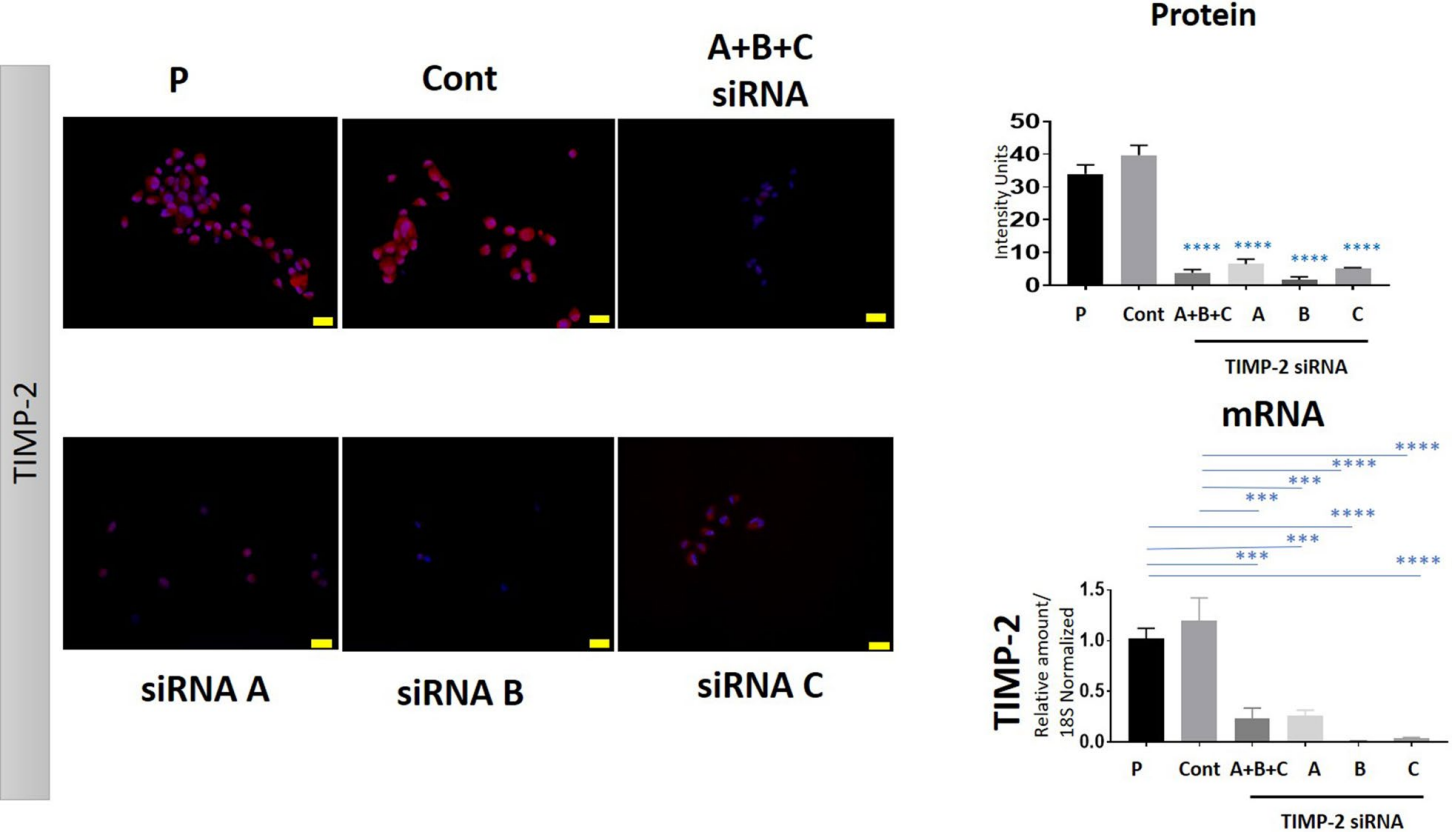
siRNA suppression of TIMP-2 in the OVCAR5 cell line. Suppression of TIMP-2 expression by siRNA transfection in the OVCAR5 cell line is described in “Methods”. TIMP-2 expression was evaluated by immunofluorescence at the protein level and at mRNA level by qRT-PCR as described in “Methods”. A, B and C are single siRNA duplexes and A + B + C is representative of a pool of all three TIMP-2 siRNAs at a 3 nM final concentration. Immunofluorescence images are representation of merged DAPI (blue) and TIMP-2 (red) staining on individual cell lines done in three passages in triplicate. The intensity of fluorescence was obtained using FIJI software. ×20 magnification; scale bar (in yellow) 20 μM; P indicates the parental cell line treated with transfection reagent only, Cont are cells transfected with scrambled siRNA. For mRNA expression, graphs represent amount of mRNA relative to 18S + SEM derived from three experiments done in triplicate. Significance was determined by one-way ANOVA and indicated by ***p < 0.001, ****p < 0.0001

We have previously described the reduction or knock down of TIMP-2 expression in ovarian cancer cell lines (OVCAR4, JOSH2, FT282) by using three distinct 27mer siRNA duplexes independently, or a pooled (A + B + C) siRNA duplexes directed against human TIMP-2 (T2-KD cells) [24]. In this study the same TIMP-2 siRNAs were used to knock down TIMP-2 expression in OVCAR5 cell line. Additional file 1: Fig. S1A indicates the location of siRNA duplexes A, B and C on Exons, 3, 2 and 1 of the TIMP-2 gene. A non-targeting siRNA was used as a Control (Cont). A relatively consistent knockdown of TIMP-2 expression at the protein and mRNA levels by single 27mer TIMP-2 siRNA duplexes or with the pooled (A + B + C) TIMP-2 siRNAs in OVCAR5 cell line compared to Cont and Parental (P) cell lines is shown in Fig. 1. TIMP-2 protein expression was reduced by ~ 90% and mRNA expression by ~ 80% in siRNA transfected cells compared to Cont and Parental (P) cell lines (Fig. 1). As both the single 27mer duplex siRNA (A or B or C) and the pooled siRNA (A + B + C) showed equivalent degrees of TIMP-2 expression at the protein and mRNA levels, the next phase of experiments was performed only with pooled TIMP-2 siRNAs (A + B + C); the cells are indicated as T2-KD cells. In this context, the use of pooled siRNAs in combination with the lowest possible amount of siRNA has been described as a preferred approach to minimize the off-target effects in other studies [27].

### Editing of TIMP-2 gene in the OVCAR5 cell line using CRISPR/Cas9 technology

The OVCAR5 cells were transfected with CRISPR assembled with two different guide RNAs (gRNA) targeting different areas in Exon 1 of the TIMP-2 gene (Additional file 1: Fig. S2A). The sequences of TIMP-2 targeted by Cas9 are shown in Table 1. The concentration that killed 50% of the cells with increasing concentration of puromycin was determined by MTT assay (IC_50_ values, Additional file 1: Fig. S2B). The puromycin resistant cells were GFP sorted on a Flow cytometer. After the first GFP sorting of the gRNA1 cell line, only ~ 3% of the cells were GFP positive, while in gRNA2, ~ 90% were GFP positive, with 0% cells GFP positive in control cells. After 10 more passages cells were re-sorted for GFP. Final sorting results showed 0% of GFP positive cells in gRNA1 and control cell lines, while the gRNA2 cell line had 100% GFP positive cells (Additional file 1: Fig. S2C). Puromycin sensitivity was checked in all cell lines (parental, CRISPR control, gRNA1 and gRNA2 cell lines). As expected, the parental cell line had the highest sensitivity to puromycin (Additional file 1: Fig. S2D). The gRNA2 cell line showed a 61-fold increase, and gRNA1, an 82-fold increase in puromycin resistance compared to parental cell line (Additional file 1: Fig. S2D). However, unexpectedly, the control CRISPR cell line showed a 37-fold increase in puromycin resistance compared to parental cell line.

**Table 1:** The sequences of TIMP-2 targeted by Cas9

Cas9-guided vector	Target Sequence (5′ → 3′)	Gene targeted	Exon
gRNA1	AGCAGCTGCAGGCGTCGGCC	TIMP-2	1
gRNA2	CGCACCCTGCGGCTGGCGCT	TIMP-2	1
Scramble control	GCACTACCAGAGCTAACTCA	None	N/A

### Reduced TIMP-2 expression in OVCAR5 CRISPR/Cas9 edited cell lines confirmed by protein analyses

**Fig. 2:**
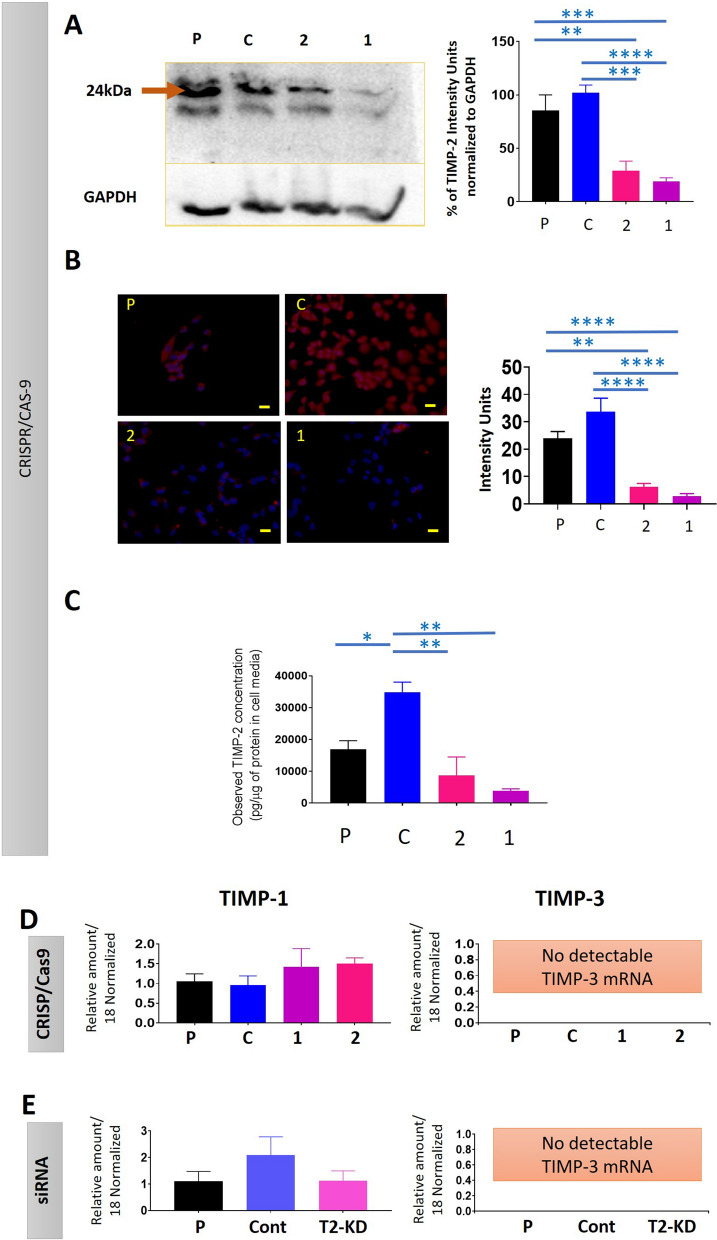
Expression of TIMP-2 and other TIMPs in response to TIMP-2 suppression by CRISPR/Cas9 and siRNA in OVCAR5 cell line. Cell lines studied are P—Parental; C—CRISPR control; 2—gRNA2, 1—gRNA1 cell lines; Cont-siRNA control, T2-KD-TIMP-2 siRNA knocked down and P, parental lipofectamine only treated control cells. **A** Expression of cellular TIMP-2 by Western blot in parental and CRISPR/Cas9 treated cell lines. Representative image of a Western blot of TIMP-2 and GAPDH proteins on the cell lysates of the respective cell lines. The additional cross-reactive bands observed are indicated in manufacture’s information. Graphs indicates densitometry intensity units of TIMP-2 protein bands normalized to GAPDH protein. The data presented is a mean of three independent experiments. Values are mean ± SEM. Significance was obtained using One-way ANOVA. **p < 0.01, ***p < 0.001, ****p < 0.0001 when compared to the control and parental cell lines. **B** Expression of cellular TIMP-2 by immunofluorescence in CRISPR/Cas9 treated, control and parental cell lines. Images are representations of merged DAPI (blue) and TIMP-2 (red) staining of individual cell lines performed in three independent experiments, in triplicate. ×40 magnification; scale bar (in yellow) 40 µM. Graphs indicates the intensity of fluorescence obtained using FIJI software as described in “Methods”. Significance was obtained using One-way ANOVA. **C** Secreted TIMP-2 protein in CRISPR/Cas9 treated, control and parental cell lines. The expression of secreted TIMP-2 in cell conditioned medium was deduced by ELISA as described in “Methods”. Results are expressed as total TIMP-2 secreted protein (pg/µg of protein in cell medium). Values are mean ± SEM and each bar graph represents n = 3 for each cell line collected in three independent experiments. Significance was obtained using One-way ANOVA *p < 0.05, **p < 0.01, and ****p < 0.0001 when compared to the control cell line and parental cell line. **D**, **E** mRNA expression of TIMP-1 and TIMP-3 in siRNA-mediated TIMP-2 suppressed cells. mRNA expression of TIMP-1 and TIMP-3 was deduced by qRT-PCR as described in “Methods”. Values are mean ± SEM and each bar graph represents n = 3 for each cell line collected in three independent experiments

The expression of TIMP-2 in the CRISPR/Cas9 edited, control and parental cell lines was evaluated by Western blot, immunofluorescence, and ELISA. Compared to either parental or CRISPR control cell lines, TIMP-2 protein levels where significantly down-regulated in both cellular and secreted protein contents in gRNA1 and gRNA2 cell lines (Fig. 2, Additional file 1: Fig. S3). TIMP-2 cellular protein measured by WB was downregulated in gRNA1 by 81% and by 72% in gRNA2 when compared to CRISPR control cell line; and by 78% and 66% respectively when compared to parental cell line (Fig. 2A). By IF, there was a 92% reduction of TIMP-2 protein in gRNA1 and an 82% reduction in gRNA2 when compared to CRISPR control cell line; and by 88% and 74% respectively when compared to the parental cell line (Fig. 2B). There was an 89% reduction of TIMP-2 secreted protein in gRNA1 and 75% reduction in gRNA2 compared to vector control cell lines in the conditioned medium as demonstrated by ELISA (Fig. 2C). When compared to the parental cell line, TIMP-2 secreted protein was downregulated by 77% in gRNA1 and by 49% in gRNA2 (Fig. 2C). All these methods demonstrated that there was on average 63% to 76% TIMP-2 protein reduction in the gRNA2 cell line, and 81% to 87% TIMP-2 reduction in the gRNA1 clone when compared to parental and CRISPR control cells, respectively. Interestingly, the CRISPR control clone had an increase of 56% in TIMP-2 protein expression (average expression across all three different experimental groups e.g., WB, IF and ELISA) (Fig. 2C). The significant reduction in TIMP-2 protein expression in both the gRNA 1 and gRNA2 cell lines measured by the three methods (summarised in Table 2), imply stable knock down of expression of the TIMP-2 gene in both gRNA1 and gRNA2 cell lines. The knock down of TIMP-2 expression by either the siRNA or CRISPR/Cas9 methods had no significant effect on the expression of TIMP-1 (Fig. 2D, E). TIMP-3 mRNA was below detection in all the OVCAR5 cell lines (Fig. 2D, E).

**Table 2:** Summary of reduction in TIMP-2 protein expression in both the gRNA 1 and gRNA2 cell lines measured by the three methods

Method (units) used	TIMP-2 expression	Parental (Mean ± SEM)	Control (Mean ± SEM)	gRNA2 (Mean ± SEM)	gRNA1 (Mean ± SEM)
WB (% of intensity units normalised to GAPDH)	Cell lysate	85.38 ± 14.64	102.0 ± 7.20	28.89 ± 9.07	18.95 ± 3.47
IF (intensity units)	Cellular	23.99 ± 2.47	33.69 ± 4.97	6.17 ± 1.30	2.85 ± 0.93
ELISA (pg/µg of TIMP-2 protein in cell media)	Secreted into the cell media	16,876 ± 2717	34,872 ± 3137	8,674 ± 5823	3,846 ± 614.5

### Expression of MMP-2 and MMP-14 in response to CRISPR/Cas9 and siRNA knock down of TIMP-2 expression

**Fig. 3:**
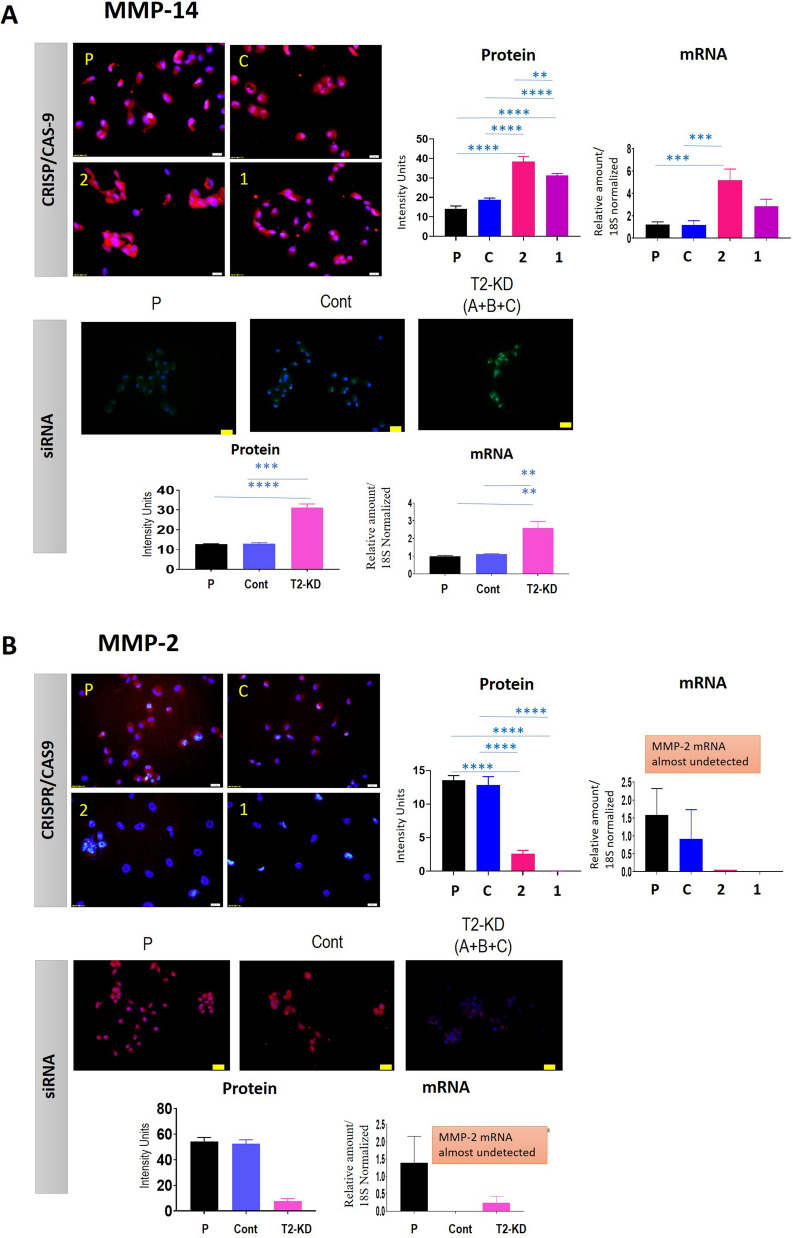
The expression of cellular MMP-14 and MMP-2 in parental, CRISPR/Cas9 and siRNA treated TIMP-2 suppressed cells. Cell lines/cells are as described in this figure. **A** The expression of MMP-14 protein by immunofluorescence and mRNA by qRT-PCR. Immunofluorescent images are representative of three independent experiments in triplicate showing merged DAPI (blue) and MMP-14 (green) staining on individual cell lines. ×20 magnification; scale bar (in white) 20 µM. For mRNA expression, values are mean ± SEM and each bar graph represents n = 3 for each cell line collected in three independent experiments. **B** The expression of MMP-2 protein by immunofluorescence and mRNA by qRT-PCR. Images are representative of three independent experiments in triplicate showing merged DAPI (blue) and MMP-2 (red) staining on individual cell lines. ×20 magnification; scale bar (in white) 20 µM. Histogram values are mean ± SEM and each bar graph represents n = 3 for each cell line collected in at least three independent experiments. For mRNA expression values are mean ± SEM and each bar graph represents n = 3 for each cell line collected in three independent experiments. Significance was obtained using One-way ANOVA **p < 0.01, ***p < 0.001, ****p < 0.0001 when compared to the control and parental cell lines

Our previous study had shown that knock down of TIMP-2 expression by siRNA in Fallopian-tube derived non-malignant (FT282) and ovarian cancer cell lines (OVCAR4 and JOSH2) showed significant upregulation of MMP-14 expression with downregulation of MMP-2 expression (both protein and mRNA) and activation [24]. In this study, we show similar upregulation of MMP-14 expression and downregulation of MMP-2 expression in CRISPR/Cas9 (gRNA1 and gRNA2 cell lines) and siRNA-mediated (T2-KD cells) knock down of TIMP-2 in the OVCAR5 cell line (Fig. 3A, B). CRISPR/Cas9-mediated knock down of TIMP-2 in the OVCAR5 cell line resulted in an enhanced expression of MMP-14 both at the protein and mRNA level in the gRNA2 cell lines when compared to control and parental cell lines. MMP-14 was also significantly upregulated at the protein level in gRNA1 cells compared to control and parental cell lines, but the upregulation at the mRNA level was not statistically significant (Fig. 3A). Consistent with the siRNA results in the previous study [24], MMP-14 expression in T2-KD OVCAR5 cells was significantly enhanced both at the protein and mRNA levels compared to control cells (Fig. 3A). In agreement with our previous study [24], MMP-2 protein expression was also reduced significantly in OVCAR5 T2-KD cells and gRNA1 and gRNA2 cell lines compared to control cells (Fig. 3B). The mRNA levels of MMP-2 were almost undetectable by qRT-PCR in both gRNA1 and gRNA2 cell lines, but limited expression was observed in parental, CRISPR control, T2-KD cells, and siRNA vector control cells (Fig. 3B). The discordance in the mRNA and protein expression of MMP-2 in response to TIMP-2 knockdown can be due to several reasons some of which has been described in the literature [27]. The mRNA synthesis of a particular protein at a given time depends on its expression and decay [28]. The half-life of MMP-2 mRNA is about 49 h [29], thus the MMP-2 gene may not be actively transcribed at the time of mRNA detection. On the other hand, the half-life of protein is expected to be much longer, suggesting that the protein expression in this case would be more evident than mRNA expression.

### Expression of EMT-associated genes in response to CRISPR/Cas9 and siRNA knock down of TIMP-2 expression

**Fig. 4:**
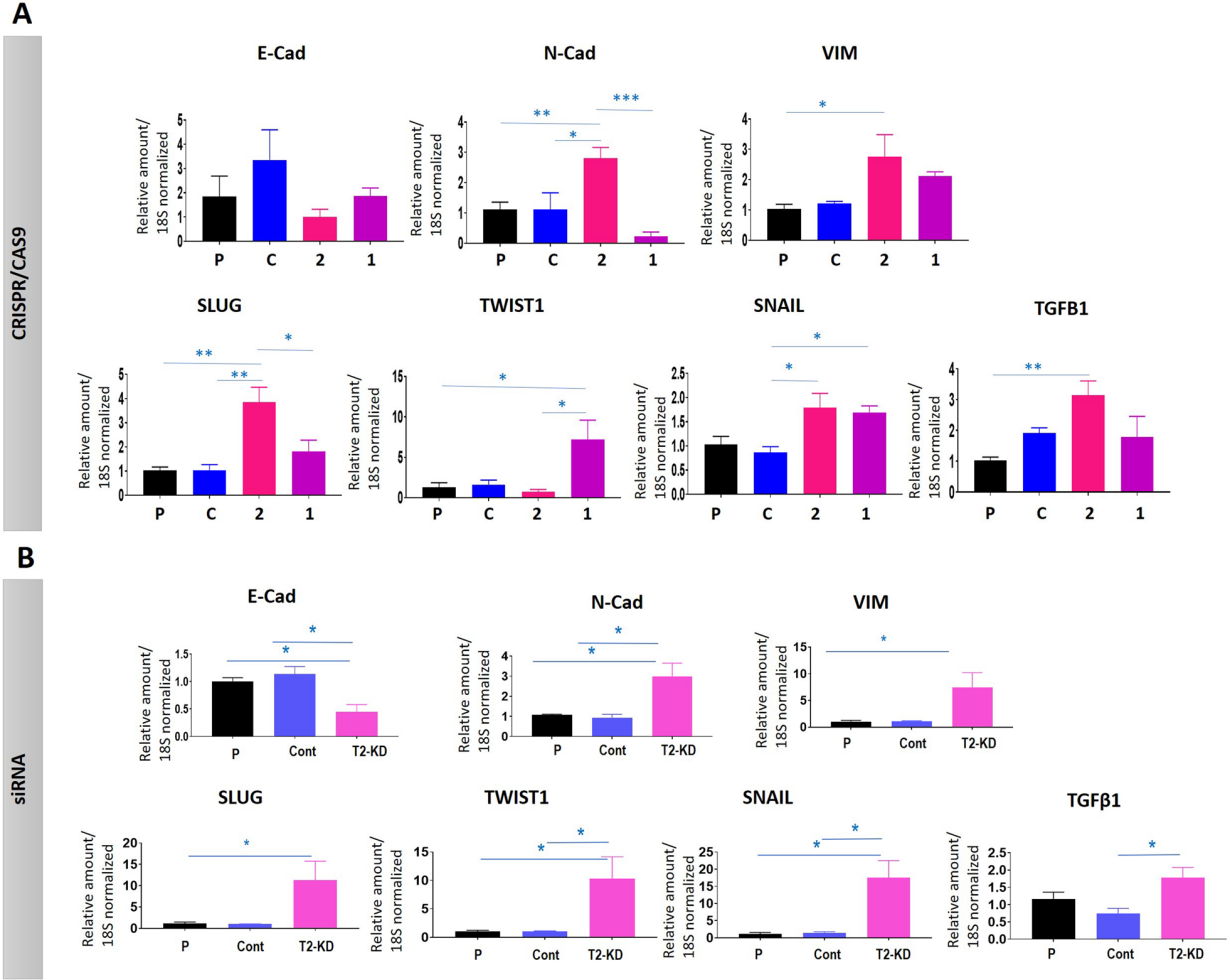
Effect of TIMP-2 suppression by **A** CRISPR/Cas9 and **B** siRNA methods on the mRNA expression of EMT-associated genes in OVCAR5 cells. The cell lines/cells are described in Fig. 3. **A**, **B** The mRNA expression of E-Cad, N-Cad, VIM, SLUG, TWIST1, SNAIL and TGFβ1 deduced by qRT-PCR. Values are mean ± SEM and each bar graph represents n = 3 for each cell line collected in independent experiments. Significance was obtained using One-way ANOVA. *p < 0.05, **p < 0.01, ***p < 0.001, ****p < 0.0001 when compared to the control and parental cell lines

Changes in the expression of TIMP-2 and certain MMPs have been shown to lead to changes in EMT genes in various cancer models [30, 31]. Consistent with that, our previous study showed enhanced expression of key EMT genes in siRNA knocked down of TIMP-2 expression in OVCAR4 and JOSH2 cell lines compared to vector control and parental cells [24]. In this study, although we show that the mRNA expression of E-Cad protein was decreased in both gRNA1 and gRNA2 cell lines when compared to CRISPR control cell lines, significance was not achieved (Fig. 4A). VIM mRNA in gRNA2 cells was significantly upregulated when compared to parental cells, and although VIM mRNA also increased in gRNA1 cells, significance was not achieved when compared to either CRSIPR control or parental cell lines (Fig. 4A). Interestingly, this upregulation of VIM mRNA was also consistent with significantly enhanced N-Cad mRNA expression in gRNA2 compared to both control and parental cell lines. However, there was a slight downregulation of N-Cad in the gRNA1 clone, but it did not reach significance when compared to parental and CRISPR control cell lines (Fig. 4A).

Since there was significant upregulation of VIM and N-cad and a downregulation of E-cad in gRNA2 compared to control cell lines, we compared the mRNA expression of other EMT transcription factors SLUG, TWIST1, and SNAIL in these cell lines (Fig. 4A). The mRNA expression of SLUG and SNAIL were significantly upregulated in gRNA2 cells when compared to CRISPR control cells, but their upregulation was not significant when compared to parental cells (Fig. 4A). While significant upregulation was not achieved in the gRNA2 cell line for TWIST1 mRNA expression when compared to either control cell lines, in the gRNA1 cell line there was a significant upregulation in TWIST1 mRNA (when compared to parental cell line) and in SNAIL mRNA when compared to CRISPR control cell line. Furthermore, TGFβ1 mRNA was only significantly upregulated in the gRNA2 cell line when compared to parental OVCAR5 cell line, but no such upregulation of TGFβ1 was observed in gRNA1 cell line compared to any control cell lines (Fig. 4A).

Consistent with the EMT-marker profile in gRNA2 cell line, knock down of TIMP-2 by siRNA in OVCAR5 cell line (T2-KD cells) showed a similar enhanced expression of VIM, N-Cad, SLUG, SNAIL, and TGFβ1 compared to parental or vector control cells (Fig. 4B).

### Proliferation, migration, and invasion of the OVCAR5 cell line in response to TIMP-2 knock down by CRISPR/Cas9 and siRNA

**Fig. 5:**
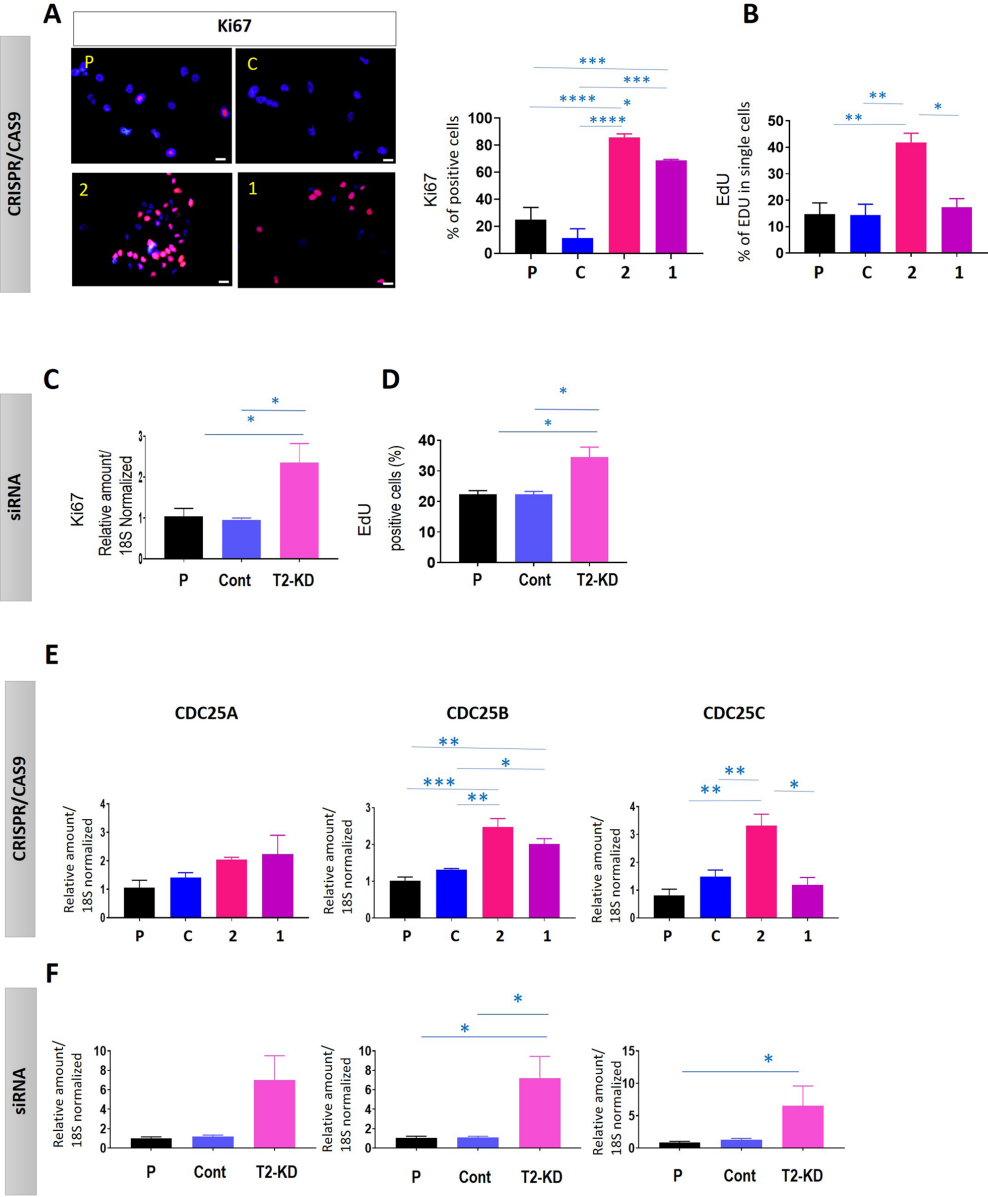
Effect of TIMP-2 suppression on proliferation. The cell lines/cells studied have been described in Fig. 3. **A**, **C** Proliferation evaluated by Ki67 staining. Immunofluorescence of Ki67 (a marker of proliferation) staining was performed as described in “Methods”. Representative images of Ki67 positive cells (red) and DAPI (blue) combined. ×20 magnification; scale bar (in white) 20 µM. **B**, **D** Proliferation evaluated by EdU staining. Cells were stained for EdU and analysed by flowcytometry as described in “Methods”. Results are expressed as % of EdU positive cells (or cells in the S-phase of cell cycle progression). **C**, **E**, **F** mRNA expression of Ki67, CDC25A, CDC25B and CDC25C were evaluated by qRT-PCR. Bar graphs are representative of three independent experiments in triplicate. Significance was obtained using One-way ANOVA and is indicated by *p < 0.05, **p < 0.01, ***p < 0.001, ****p < 0.0001

**Fig. 6:**
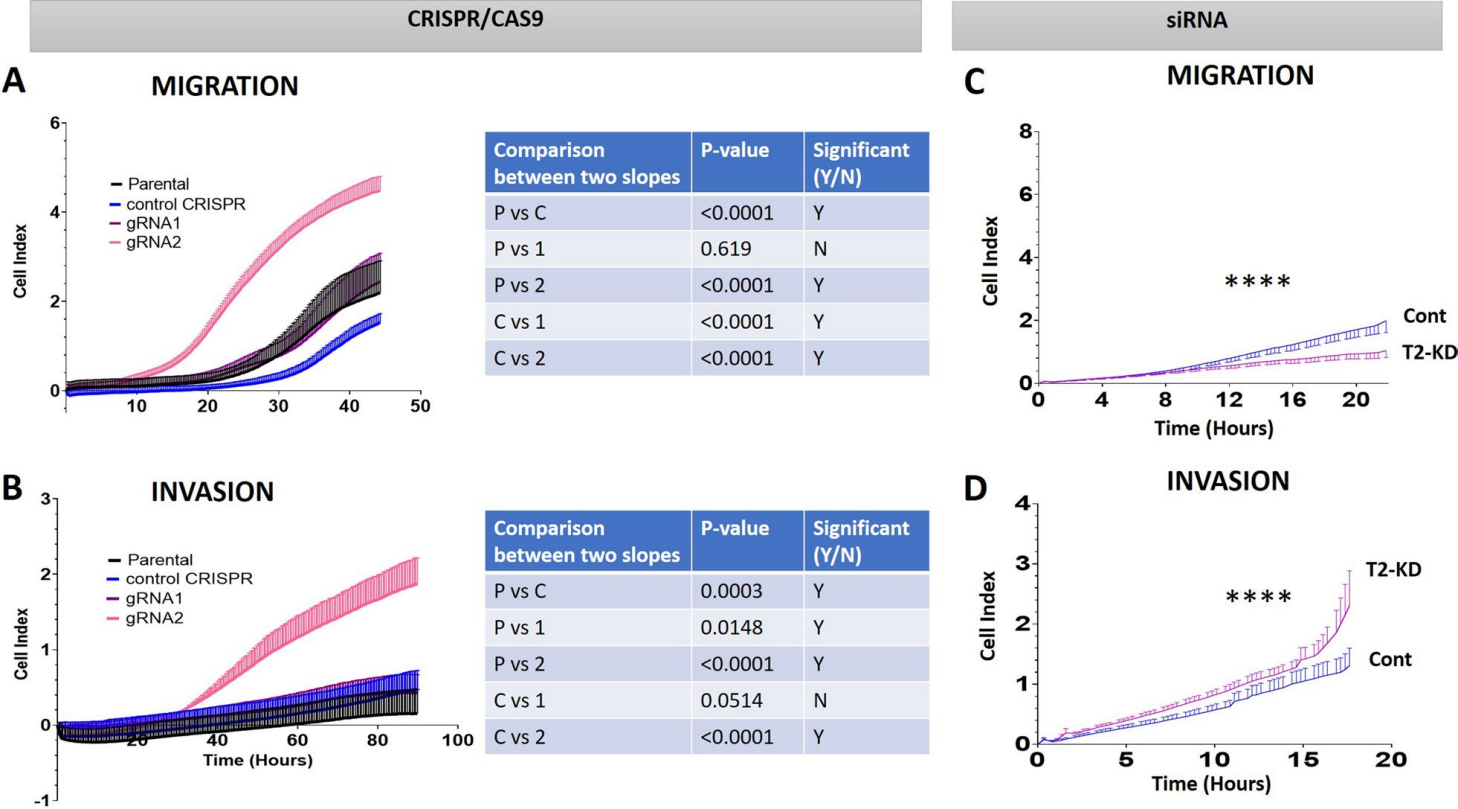
Effect of TIMP-2 suppression on migration and invasion. The cell lines studied were gRNA1 (1), gRNA2 (2), CRISPR control (C), parental (P) and pooled siRNA (A + B + C) TIMP-2 suppressed (T2-KD), vector control (C) and parental (P, parental cell line treated with lipofectamine) cells. **A**, **C** Migration and **B**, **D** invasion were assessed by xCELLigence real-time cell analysis. For assessment of invasion, the electrodes were coated with Matrigel and the bottom of the well contained reduced serum medium (OPTI-MEM). Significance was assessed by linear regression analyses of the slopes and results are shown in the tables next to each graph. Significance obtained using One-way ANOVA and is indicated by ****p < 0.0001

The proliferation of gRNA1 and gRNA2 cells compared to CRISPR control and parental cell lines, and T2-KD cells compared to vector control and parental cells, were analysed using mRNA analysis or staining for Ki67, and by measuring EdU incorporation into DNA using flow cytometry (Fig. 6, Additional file 1: Fig. S3). Both assays revealed a significant enhancement in cellular proliferation in gRNA2 cells compared to CRISPR control and parental cells (Fig. 5A, B). However, only Ki67 positive cells were significantly enhanced in the gRNA1 cell line when compared to CRISPR control and parental cell lines; no significant difference was achieved by measuring EdU in the gRNA1 cell line compared to control (Fig. 5A, B). Consistent with the proliferation results in the gRNA2 cell line, both mRNA levels of Ki67 and EdU staining showed significant upregulation of proliferative capacity of siRNA transfected T2-KD cells compared to vector control and parental cells (Fig. 5C, D).

The mRNA expression of cell cycle enzymes, CDC25A, CDC25B and CDC25C were evaluated after suppression of TIMP-2 expression to further characterize the proliferative capacity of gRNA1, gRNA2 cell lines and T2-KD cells in relation to control and parental cells. The mRNA expression of CDC25B and CDC25C were significantly upregulated in gRNA2 cells, while only the CDC25B gene was upregulated in the gRNA1 cells compared to control and parental cell lines (Fig. 5E). Consistent with the gRNA2 cell line, the mRNA expression of both CDC25B and CDC25C were significantly enhanced in T2-KD cells compared to control cells (Fig. 6F). There was, however, no change in the mRNA expression of CDC25A gene in any of the OVCAR5 CRISPR/Cas9 or T2-KD cells compared to either control or parental cells (Fig. 5E, F).

Migration and invasion properties of gRNA1, gRNA2, T2-KD, and CRISPR and vector control OVCAR5 cells lines under monolayer conditions were assessed by xCELLigence Real Time Cell Analysis. Both gRNA1 and gRNA2 cell lines had significantly enhanced migration compared to the CRISPR control cell line (Fig. 6A). However, only the migration of the gRNA2 cell line was significantly upregulated when compared to the parental OVCAR5 cell line (Fig. 6A). There was no significant increase in migration of the gRNA1 cell line compared to the parental line. Interestingly, the CRISPR control cell line migrated significantly slower than the parental cell line (Fig. 6A).

Both gRNA1 and gRNA2 cell lines revealed a significant enhancement of invasion through Matrigel when compared to the OVCAR5 parental cells under monolayer conditions. More specifically, gRNA2 demonstrated a greater invasion than gRNA1 (Fig. 6B). However, comparison between CRISPR control and gRNA1 invasion showed no significant difference (p = 0.0514) but a very large statistical difference in the slope curves was observed between the gRNA2 and CRISPR control cell lines (Fig. 6B). There was a significant difference between parental OVCAR5 and CRISPR control cell lines, with the CRISPR control being more invasive (Fig. 6B). Contrary to that, T2-KD cells had significantly higher invasion compared to control cells, but the migration of T2-KD cells was significantly reduced compared to control cells (Fig. 6C, D).

### Chemosensitivity of the OVCAR5 cell line in response to TIMP-2 knock down by CRISPR/Cas9 and siRNA

**Fig. 7:**
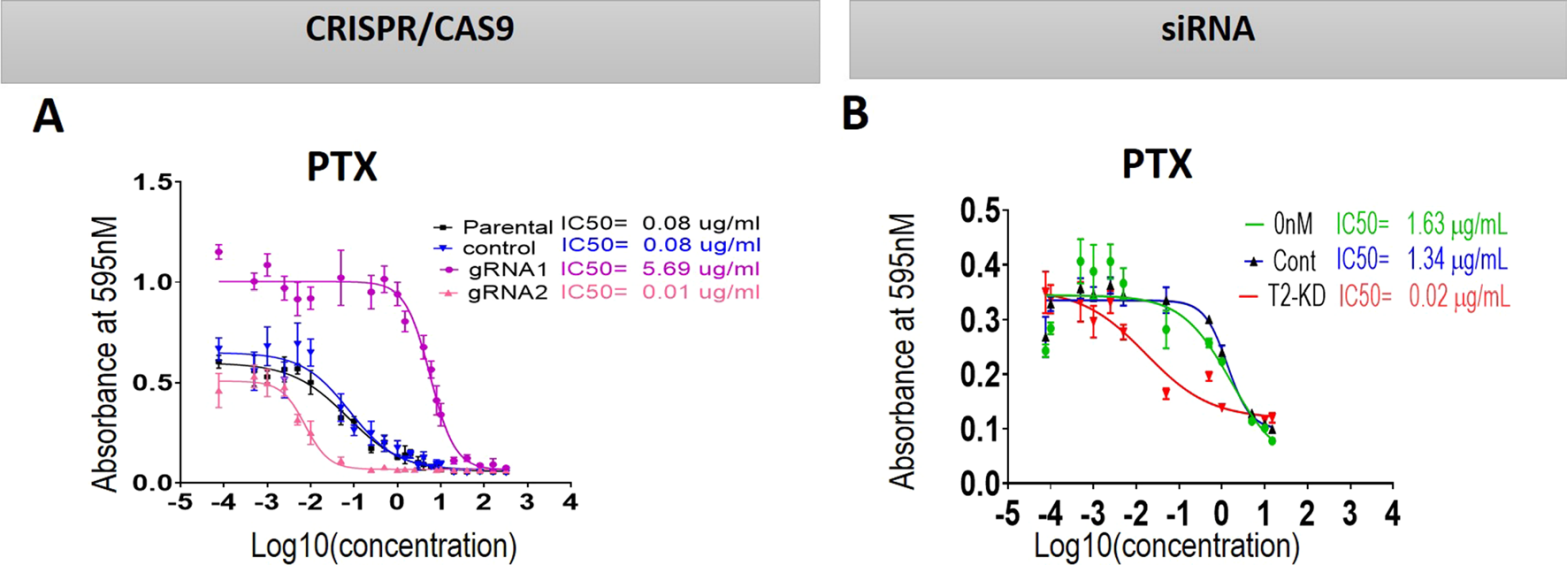
Effect of paclitaxel (PTX) on TIMP-2 knocked down CRISPR/Cas9 and siRNA transfected cells and their controls. **A**, **B** The cell lines/cells are described in the legend for Fig. 3. Cell lines were treated for 48 h with varying concentrations of PTX, and the IC_50_ values (the concentration that kills 50% of the cells) were determined by MTT assay. Graphs are representative of three independent experiments done in triplicate

**Fig. 8:**
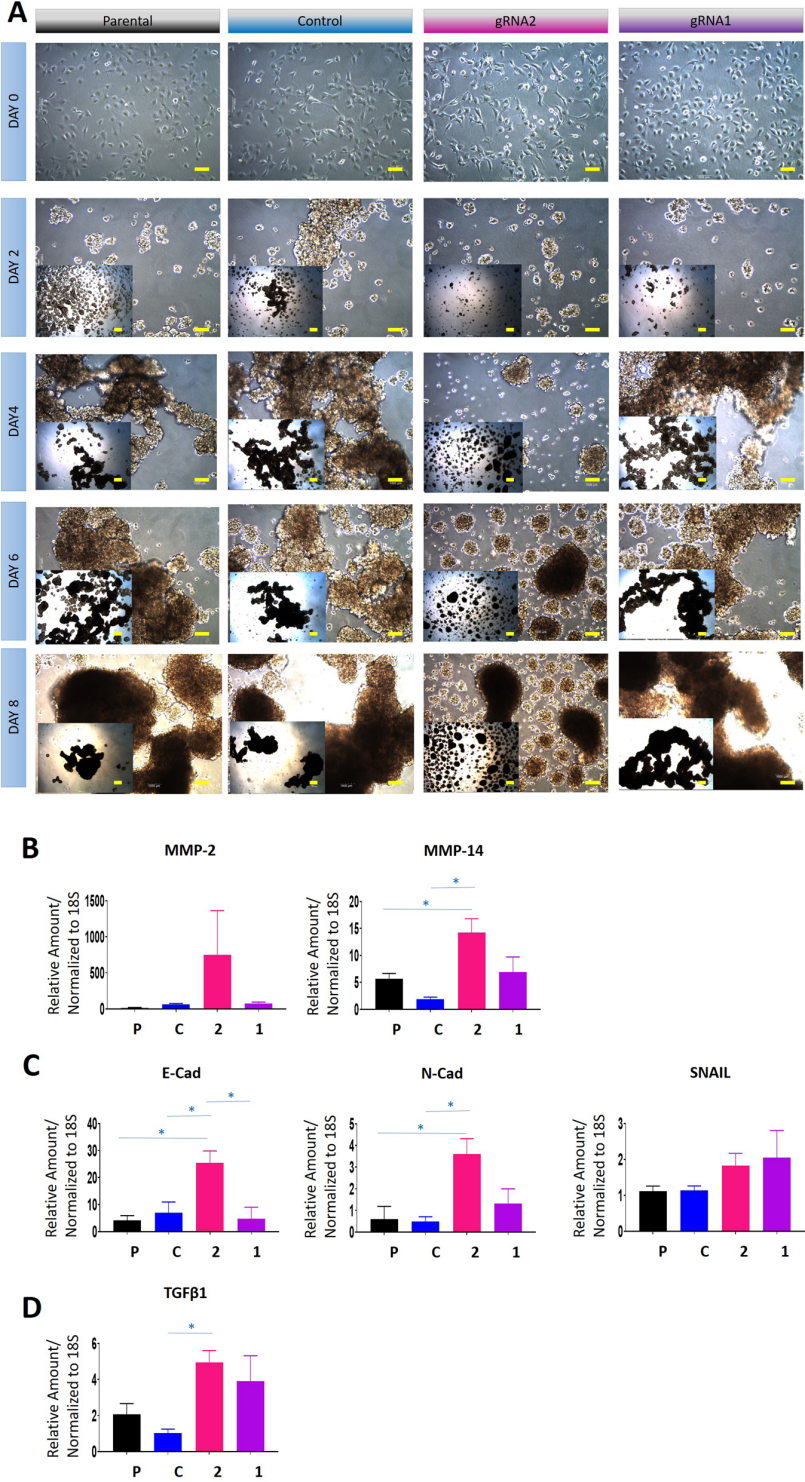
3D spheroid growth and mRNA expression of EMT-associated genes in CRISPR/Cas9 edited cell lines and their controls. The cell lines studied are: Parental (P), CRISPR control (C), gRNA2 (2) and gRNA1 (1). Spheroid formation on low attachment plates was monitored using light microscopy. **A** Images of spheroids every second day from day 0 to day 8. Photographs are of ×4 magnifications. **B**–**D** The mRNA expression of MMP-14, MMP-2, E-Cad, N-Cad, SNAIL and TGFβ1 deduced by qRT-PCR as described in “Methods”. Values are mean ± SEM and each bar graph represents n = 3 for each cell line collected in independent experiments. Significance was obtained using One-way ANOVA. *p < 0.05, when compared to the control and parental cell lines

We have previously shown that knock down of TIMP-2 in T2-KD ovarian non-malignant (FT282) and cancer cells (OVCAR4 and JOSH2) had enhanced sensitivity to PTX compared to their matched controls [24]. In the CRISPR/Cas9 TIMP-2 edited cell lines, gRNA1 and gRNA2, knock down of TIMP-2 demonstrated different sensitivities to PTX when compared to either control or parental cells (Fig. 7A). There were no significant differences between the IC_50_ values of CRISPR control and parental cell lines, however there was a significant increase in the sensitivity to PTX in the gRNA2 cell line where the IC_50_ value was reduced by 11-fold when compared to either control or parental cell lines (Fig. 7A). However, surprisingly, there was an increase in resistance to PTX in gRNA1 cell line, as its IC_50_ value was 71-fold higher the either control or parental cell lines (Fig. 8A). Consistent with gRNA2 results, the sensitivity of OVCAR5 T2-KD cells was 60-fold less than in vector control and parental cells (Fig. 7B).

### Spheroid formation, proliferation, and reattachment of monolayer cells in response to TIMP-2 knock down by CRISPR/Cas9

The abilities gRNA1 and gRNA2 cell lines to form spheroids, proliferate and reattach in monolayer culture was tested. gRNA2 cells formed round compact spheroids compared to long elongated sheet-like spheroids formed by gRNA1 by day 8 (Fig. 8A). The spheroids in both control and parental cell lines resembled those observed with gRNA1 cells as they formed very elongated cell aggregates.

Next, we assessed the mRNA expression of MMP-2 and 14 and EMT associated genes in spheroids after 8 days in culture. The mRNA expression of MMP-14 was significantly upregulated in gRNA2 spheroids compared to parental and control spheroids, although no significant mRNA changes in MMP-2 expression were noted in gRNA2 spheroids (Fig. 8B). Both E-Cad and N-Cad were significantly upregulated in the gRNA2 spheroids when compared to parental and CRISPR/Cas9 control spheroids (Fig. 8C). There were no significant changes in the mRNA expression of transcription factor SNAIL in any of the spheroids. Since TGFβ1 has been reported to induce EMT in cancer cells, this cytokine was also studied in spheroids on Day 8, and it was found that TGFβ1 mRNA was significantly upregulated in gRNA2 cells compared to control (Fig. 8D). Although TGFβ1 mRNA levels increased in gRNA1 spheroids, this did not reach statistical significance. No significant changes in the mRNA levels of MMP-14, MMP-2, E-cad, N-cad and SNAIL were noted in Day 8 spheroids of gRNA1 compared to control cells (Fig. 8D).

**Fig. 9:**
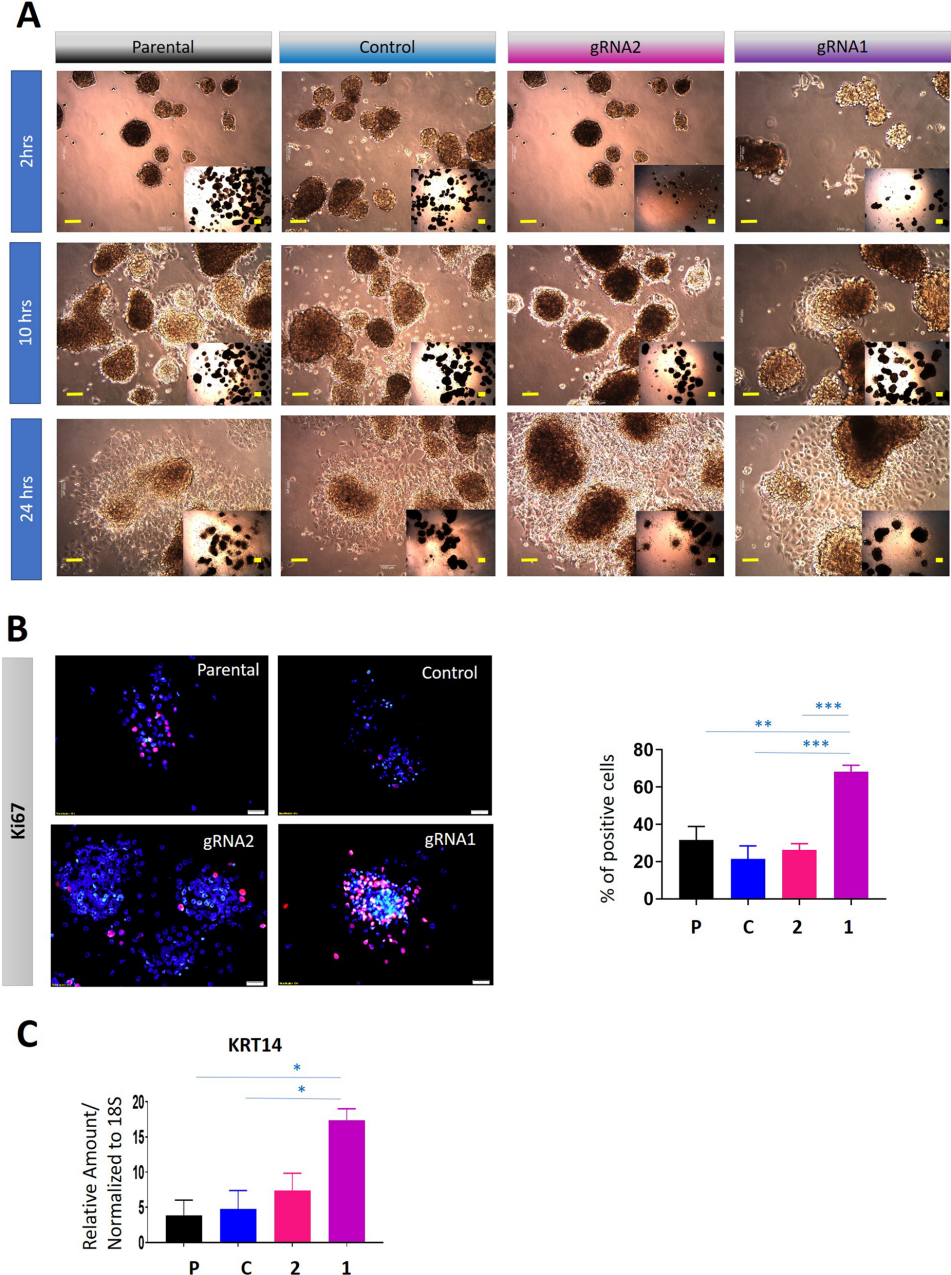
Reattachment of gRNA1 and gRNA2 cell-derived spheroids and their respective controls as monolayers; Ki67 proliferation and expression of KRT14 in reattached spheroid cells. The cells studied are described in this figure. **A** Spheroids of gRNA1 and gRNA2 cells were obtained by growth on low attachment plates. After day 10, spheroids were transferred to normal attachment plates and their attachment and spread as monolayer cultures were microscopically imaged at 2, 10 and 24 h. Scale bar (in yellow) 1000 µM. **B** Immunofluorescence of Ki67 stained cells was performed on Day 10 spheroids after 24 h of attachment as described in “Methods”. Images are representative of Ki-67 (red) and DAPI (blue) combined. ×20 magnification; scale bar (in white) 20 µM. Bar graphs represent mean ± SEM of 3 independent experiments. **C** mRNA expression of the invasive marker KRT14, evaluated by qRT-PCR in Day 10 spheroids after 24 h attachment as a monolayer. Values are mean ± SEM and the bar graph represents n = 3 independent experiments for each cell line spheroids collected. All Statistical significances were obtained using One-way ANOVA and are indicated by *p < 0.05, **p < 0.01, ***p < 0.001

The rate of attachment of gRNA1 and gRNA2 cell lines during culture for 8 days was assessed (Fig. 9A). All cell lines forming spheroids showed attachment to the normal standard plates, and by 10 h demonstrated attachment with cell migration occurring from the edge of attached spheroids (Fig. 9A). The proliferation of these spheroids was determined by Ki67 staining. Significantly enhanced proliferation was observed in the gRNA1 cell line compared to control and parental cells. The gRNA2 cell line, however, did not show significantly different Ki67 staining when compared to either control or parental spheroid cells (Fig. 9B).

Not only did the gRNA1 cell line in a spheroid-mode showed a significantly greater proliferation than gRNA2, control and parental cell lines, it also expressed significantly higher levels of the basal epithelial invasive marker, KRT14 (Fig. 9C), which has previously been found to be a determinant of invasive potential of leader cells in ovarian cancer [32], indicating that spheroids derived from gRNA1 cell line to be more invasive than spheroids arising from gRNA2, CRISPR control, and parental cell lines. There was no significant difference in the KRT14 mRNA levels between CRISPR control and parental, or between gRNA2 and either control or parental cell lines.

### The tumorigenic potential of gRNA1 and gRNA2 cells xenotransplanted into Balb/c nude mice

**Fig. 10:**
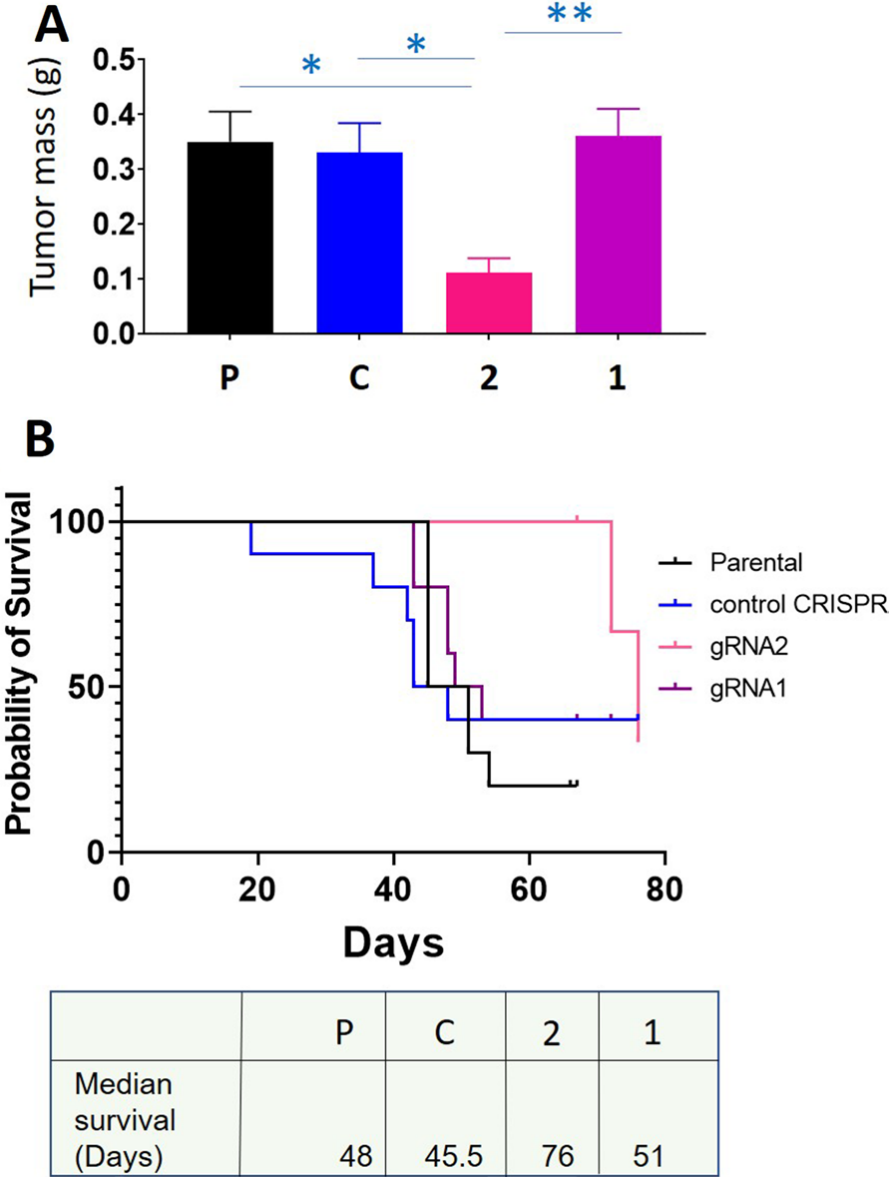
Tumour burden and Kaplan Meier Survival curves of mice injected IP with TIMP-2 CRISPR/Cas9 edited gRNA1 and gRNA2 cells and their respective controls. **A** Tumour burden obtained from mice injected IP with TIMP-2 CRISPR/Cas9 edited gRNA1 and gRNA2 cell lines and their control counter parts. Five × 106 cells of each cell line were injected IP into each mouse, n = 10 mice/group. The graph demonstrates the average tumour burden obtained from the mice in each group. Values are mean ± SEM, significance was deduced using one-way ANOVA *p > 0.05; **p < 0.01 compared to Control and Parental cell lines. **B** Kaplan Meier survival curves of mice injected with the respective cell lines as indicated above. Median survival of mice treated is shown in the Table. Significance (p < 0.05; Chi square 7.818; df 3) between the survival curves were deduced by using a Gehan–Breslow–Wilcoxon test

OVCAR5 parental, CRISPR control, gRNA1 and gRNA2 cell lines were injected IP in mice. Nine out of ten mice injected with either control or parental cell lines developed tumours located in the mesentery and omentum. However, after administration of the gRNA2 cells, six out of ten mice developed tumours, while four mice had no obvious tumours at the time of culling. In contrast, after administration of the gRNA1 cells, seven out of ten mice had small and big tumours and three mice had no obvious tumours at the time of cull. Figure 10A represents the tumour burden and Fig. 10B demonstrates Kaplan Meier survival curves for each treatment group of mice. Overall, there appeared to be a similar tumour burden in mice loaded with gRNA1 cells compared to control and parental cell lines treated mice. The median survival time for the gRNA2 group (76 days) was greater than the gRNA1 group (51 days), and both were greater than the control (45.5 days) and parental cell lines (48 days). An overall curve comparison analysis using a Gehan–Breslow–Wilcoxon test revealed that the survival curves are significantly different (p < 0.05; Chi square 7.818; df 3).

Ascites was present in three out of ten mice in the control and parental groups. No ascites was present in any of the mice injected with gRNA2 cells. Interestingly, with mice injected with gRNA1 cell lines, four out of ten had ascites. The volume of ascites found in the mice injected with gRNA1 cell line (0–200 µL) was greater that control (0 to < 20 µL) and parental (0–100 µL).

**Fig. 11:**
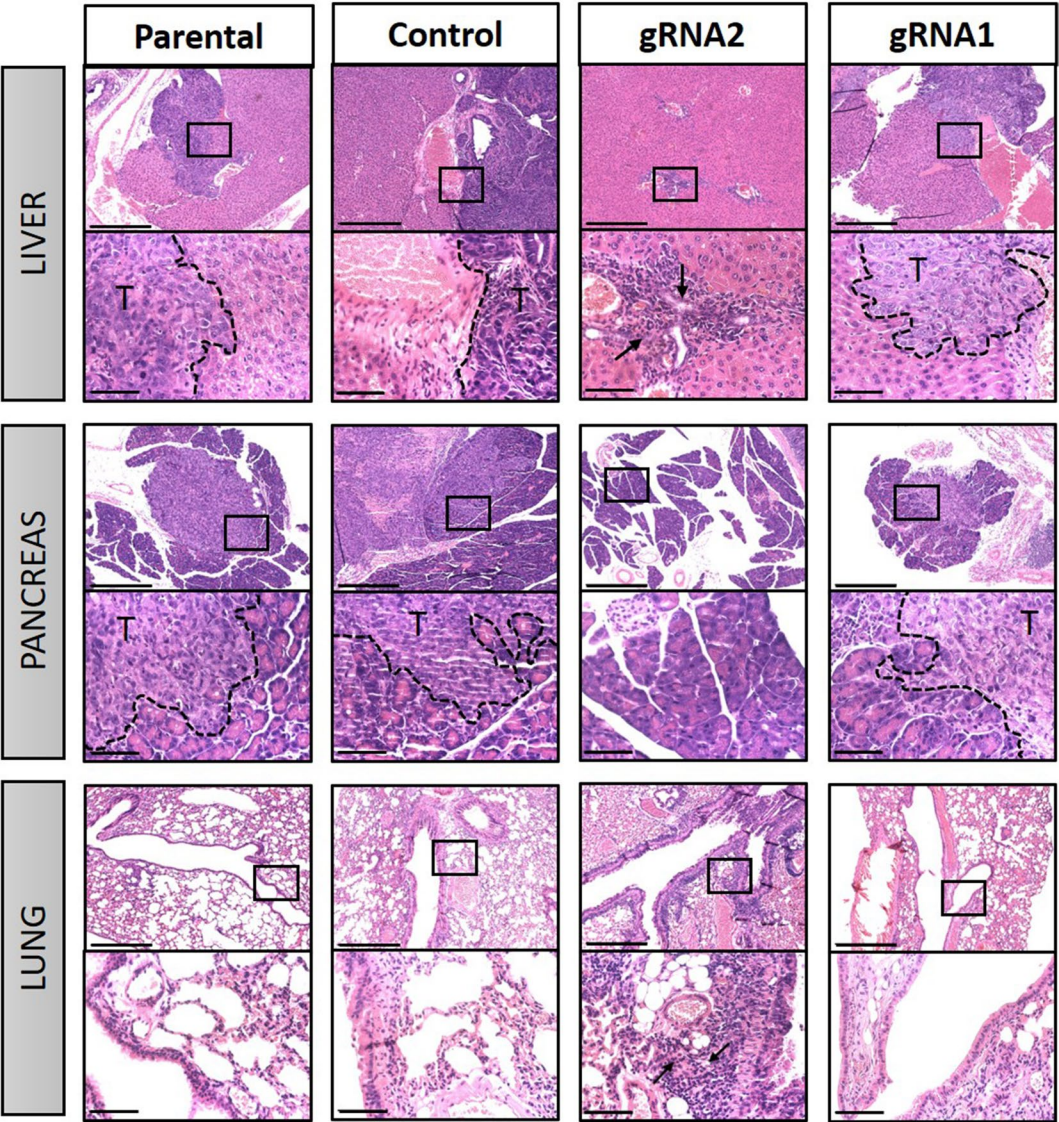
H&E staining of organs from mice injected IP with parental, CRISPR control, gRNA1 and gRNA2 cells. Tumour infiltration into liver and pancreas after IP injection of parental, CRISPR control, gRNA1 and gRNA2 cell lines is demonstrated. The infiltrating front of the tumour in each case is highlighted with a black dotted line and the tumour area within each organ is indicated by T. No infiltration of tumours was observed in lungs. Top panels of each organ are ×10 magnification and black scale bar is 500 µm; bottom panels are ×40 magnification (of the square area in the ×10 image) and yellow scale bar is 75 µm. No infiltration of tumour was observed in mice injected with gRNA2 cells. However, apparent inflammatory cells in liver and lungs of mice injected with gRNA2 cells were evident and is indicated by black arrows

H&E staining of the organs obtained from mice after dissection indicated that there was tumour invasion in organs such as the liver and pancreas by the parental, control and gRNA1 cells, but no such organ invasion was observed in the mice injected with gRNA2 cells (Fig. 11). Interestingly, portal inflammation within the liver and peri bronchial inflammation within the lungs was noted on sections from mice injected with the gRNA2 cells (Fig. 11), which was not observed in either the parental, control or gRNA1 groups. As host immune reaction is indicated by the infiltration of immune cells in specific organs, portal and bronchial inflammation may be indicative of the infiltration of proinflammatory immune cells (neutrophils, macrophages, T-helper cells, etc.) in these organs. This may suggest an increased immune response in relation to tumour development in the group of mice injected with gRNA2 cells.

**Fig. 12:**
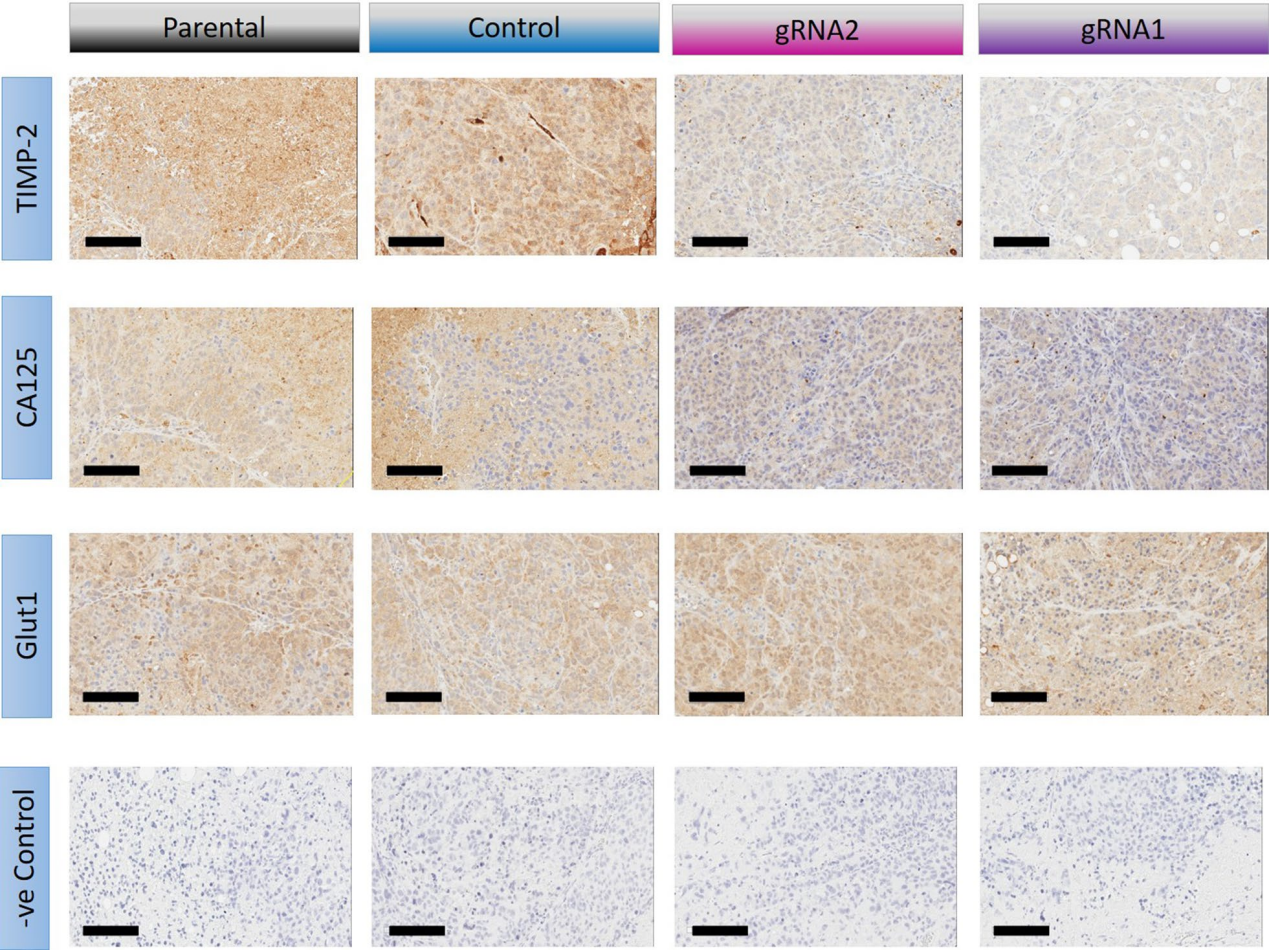
Immunohistochemical analyses of tumours obtained from mice injected IP with parental, control, gRNA1 and gRNA2 cell lines. Xenografts were evaluated for TIMP-2, CA125, Glut1 and negative (−ve) control staining as described in “Methods”. Black scale bar is 50 µm

Immunohistochemistry was performed to examine if the tumours collected from mice injected with parental, control, gRNA1 or gRNA2 cells retained some of the initial characteristics of the injected cells (Fig. 12). Xenografts obtained from mice injected with either gRNA2 or gRNA1 cells showed a reduction in the amount of TIMP-2 expression when compared to xenografts obtained from mice injected with control and parental cell lines. The expression of the CA125 tumour marker was also apparent in these xenografts (Fig. 12), consistent with their ovarian cancer origin. As high expression of GLUT1 (Glucose transporter 1) has previous been linked with high-grade undifferentiated ovarian tumours and those responding to chemotherapy [33], we investigated the expression of GLUT1 in these tumours. There was no apparent difference in the expression of GLUT1 between the groups of tumours, indicating that the knock down of TIMP-2 in ovarian tumours may not be connected to GLUT1 mediated pathways. Overall, the staining of these tumours indicates that TIMP-2 expression was reduced in the tumours originating from gRNA1 and gRNA2 cells, compared to parental and control tumours and these tumours were of human ovarian cancer origin (express CA125) (Fig. 12).

**Fig. 13:**
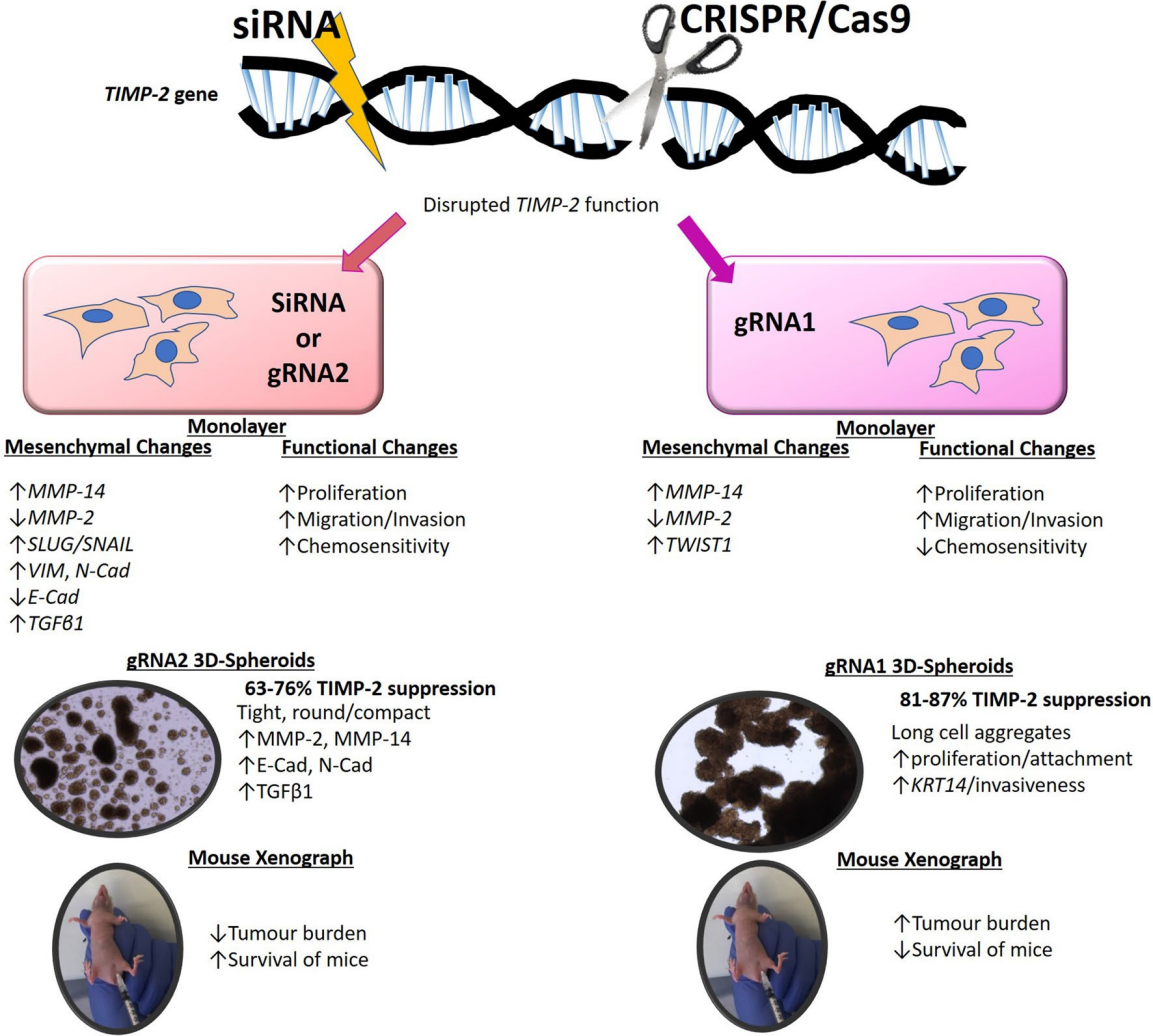
An in vitro and in vivo summary of the effects of disruption of TIMP-2 expression by either siRNA or CRISPR/Cas9 in OVCAR5 ovarian cancer cell line. Suppression of TIMP-2 expression by 60–76% in T2-KD cells and gRNA2 cell line resulted in EMT-associated changes (decreased expression of E-cad and MMP-2 with corresponding increased expression of MMP-14, SLUG, SNAIL, VIM, N-cad and TGFβ1). There was also increased proliferation, invasion and chemosensitivity to PTX. In 3D spheroid cultures, gRNA2 cells formed tight, round, and compact cell aggregates, that overexpressed MMP-2, MMP-14, E-Cad, N-Cad and TGFβ1. However, when injected into mice they produced a small tumour burden with no tumours infiltrating peritoneal organs and the mice had higher survival rates compared to control and parental cell lines. On the other hand, in gRNA1, edited by CRISPR/Cas9 for TIMP-2 gene, resulted in the inhibition of TIMP-2 expression by 81–87%. These cells exhibited low MMP-2 expression, and high MMP-14, TWIST1 and SNAIL expression, enhanced proliferation, migration, and invasion compared to control cell lines. However, this cell line was resistant to PTX and in 3D spheroid cultures formed long sheath-like cell aggregates, that had enhanced proliferation and expression of invasion marker, KRT14. Furthermore, when injected into mice gRNA1 cells produced a high tumour burden, which infiltrated peritoneal organs such as liver and pancreas and had similar survival rates when compared to controls. These data suggests that the differences in the ratios of TIMP-2 and MMPs may be critical in controlling the tumorigenic functions of ovarian cancer cells

A summary of functional changes induced by TIMP-2 expression suppression by siRNA and CRISPR/Cas9 in OVCAR5 ovarian cancer cell line in vitro culture and in a in vivo mouse model are summarised in Fig. 13.

## Discussion

In the last few years substantial progress has been made in understanding the tumorigenic roles of MMPs and TIMPs in cancer [9, 34]. Our previous studies have shown that the expression of TIMP-2 and TIMP-3 are significantly higher in high-grade serous ovarian tumours compared to benign tumours of the same origin [16]. We have also shown diverse expression of TIMP-1, TIMP-2 and associated MMPs in ascites and ovarian cancer cell lines, and changes in the expression of these proteins in response to chemotherapy treatments [8, 16, 24]. Our recent study has shown that loss of TIMP-2 expression by siRNA affects ovarian cancer cell functions and chemosensitivity [24]. In this study by using two powerful loss of gene expression/function techniques, siRNA and CRISPR/Cas9, we compared changes in cell function by the loss of TIMP-2 expression in the OVCAR5 ovarian cancer cell line. The study demonstrates that even though both techniques knocked down TIMP-2 expression by 70–90% of the vector control cells in OVCAR5 cell line, the functional outcome of siRNA generated transfectant, T2-KD cells, functionally resonated with CRISPR/Cas9 generated gRNA2 cell line. However, distinct variations in functional outcomes related to in vitro chemosensitivity, and in vivo tumour burden and metastatic dissemination in peritoneal organs existed between CRISPR/Cas9 edited gRNA2 and gRNA1 cells which had different levels of TIMP-2 suppression. These findings suggests that the degree of TIMP-2 expression may differentially alter the diverse biological functions related to ovarian cancer metastasis and chemoresistance, two major factors responsible for the high mortality in patients.

siRNAs are non-coding double-stranded RNA molecules (20–30 base pairs long) that target a particular RNA of interest resulting in transient post-transcriptional gene silencing [35]. CRISPR/Cas9, on the other hand, is a relatively new technique which edits ‘the gene of interest’ under the control of guide RNAs (gRNAs) targeting gRNA specific complementary DNA for cleavage by constitutive activation of Cas9 endonuclease [19–21]. Even though the technique was initially designed for total knock out of genes, recent literature suggest that may not be the case in many instances and off target side effects resulting from insertion–deletion (INDEL) of genome editing during the DNA repair process following Cas9 cleavage is common [20, 21, 36].

The TIMP-2 CRISPR/Cas9 system used in this study consisted of two gRNA sequences, gRNA1 and gRNA2, each targeting distinct areas of Exon 1 of the TIMP-2 gene. The system also included a linear donor fragment incorporating GFP and puromycin (expressed under the EF1A constitutive promoter) and 2A self-cleaving peptide). Under these conditions, both GFP and puromycin should be incorporated into the target gene by transcription, either in the forward or reverse direction, and the resultant cell lines should have interruption of TIMP-2 expression, puromycin resistance and expression of GFP. However, the lack of GFP in the gRNA1 cells was not predicted and is difficult to explain; it may have occurred due to lack of or deficiency in the transport of donor fragment (GFP in this case) and its associated transcription in the cells. It can be postulated that integration of GFP during CRISPR/Cas9 editing, especially in the reverse direction, may modify the structure of local chromatin, producing de novo methylation at the GFP integration site after repair, which may inhibit the transcription of GFP expression through the EF1A promoter [37, 38]. Alternatively, as both GFP and puromycin donors are packaged and transcribed into the recipient cells, there is a possibility that the donor GFP in the gRNA1 cells may have only partially integrated or may not have been integrated at all resulting in the void of GFP expression in gRNA1.

Whatever the status of the GFP expression, both gRNA1 and gRNA2 cell lines showed reduced expression of cell bound as well as secreted TIMP-2. Overall, there was on average 63% to 76% TIMP-2 protein reduction in the gRNA2 cells, and 81% to 87% TIMP-2 reduction in the gRNA1 cells when compared to parental and CRISPR control cells, respectively. Interestingly, the 56% increase in TIMP-2 protein expression (average expression) in CRISPR control cells again highlights the potential off-target effects of the CRISPR/Cas9 technique [36, 39].

Further to that, puromycin selection may also create a toxic microenvironment installing an abnormal behaviour in the recipient surviving cells. In that context, the puromycin-resistant lentiviral control shRNA vector (pLKO.1) has been shown to induce an unexpected cellular differentiation of P19 embryonic stem cells [40]. In addition, puromycin efficiently suppressed cancer stem cell states in tumour-spheres and monolayer cultures [41], and prevented the capacity of breast cancer cells to form micro-tumours in non-adherent, non-differentiating conditions [41, 42]. Thus, it is likely that incubation in puromycin may have affected the behaviour of the OVCAR5 cell line, which may have been accentuated in the CRISPR control cells resulting in greater resistance to puromycin and significantly enhanced expression of secreted TIMP-2 compared to the parental cell line. Nevertheless, these cells were used for comparison but always including parental (untransfected and puromycin-free) OVCAR5 cells as a baseline.

In this study the efficiency of the CRISPR/Cas9 technique was compromised to a large extent by the difficulty in purifying CRISPR/Cas9 edited clones by single cell selection. OVCAR5 cell line is dependent on cell-to-cell contact for growth and sustenance. This cell line normally is passaged at a split ratio of 1:3 to 1:6 and is not sustainable at lower dilutions (more than 1:10). Hence, selection of a single cell clone by limiting dilution after CRISPR/Cas9 transfection and puromycin selection was not possible. As a result, there is likely to be a mixture of homozygous and heterozygous clones of TIMP-2 suppressed cells in gRNA1 and gRNA2 cell lines. To produce a total suppression of the gene, both alleles needed to be edited in a single cell (producing a homozygous deletion). Nevertheless, the cell line (OVCAR5), was selected because of its in vivo tumorigenic capacity when compared to other serous ovarian cancer cell lines which are often difficult to grow in mice [43, 44].

In our previous study we have shown that knockdown of TIMP-2 by siRNA resulted in the induction of an EMT-like process in ovarian cancer cell lines (OVCAR4 and JOSH2) [24]. We now show an induction of EMT and significant changes in EMT-related genes (suppression of mRNA expression of E-Cad, upregulation of mRNA expression of mesenchymal genes such as VIM, N-Cad, SLUG and SNAIL) by the knock down of TIMP-2 by siRNA in T2-KD OVCAR5 cells and in the gRNA2 cells. We also show that the EMT phenomenon in T2-KD and gRNA2 ovarian cancer cells may be mediated by a significant upregulation of TGFβ1 expression [45]. However, when TIMP-2 protein levels were suppressed in the gRNA1 cells, there was no significant increase in the level of TGFb1 expression but instead, a significant mRNA upregulation of EMT-associated TWIST1 was observed which was non-existent in gRNA2 cell line. These results suggest that CRISPR-mediated knock down of TIMP-2 expression in gRNA2 cell line may induce an EMT-like profile through the induction of TGFβ1-mediated signalling pathway. However, a similar EMT-mediated process may have been induced in gRNA1 cell line through activation of TWIST1 transcription factor. However, the cytokines/growth factors mediating this induction of EMT associated genes yet remains to be determined. Consistent with this finding, enhanced expression of TWIST protein and mRNA expression has been reported in patients diagnosed with invasive ductal carcinomas with shorter progression-free survival [46]; TWIST-mediated EMT in breast cancer cells have been shown to predict poor prognosis in breast cancer patients [47]; promote invasion, metastasis and therapeutic resistance through cancer stem cell phenotype [48]. In that context, it should also be indicated that ovarian cancer cells can exist in an intermediate ‘hybrid E/M’ state or ‘within a EMT spectrum’ with characteristics of both epithelial and mesenchymal cells, depending on the stimulus they receive from the TME [49–53]. In line with this theory, it has been postulated that, epithelial cells express high E-Cad and low VIM because they have low TGFβ, while mesenchymal cells express low E-Cad and high VIM and have high TGFβ. However, hybrid E/M cells express both high E-Cad and high VIM and a non-significant change in levels of TGFβ1 [50]. These observations may resonate with the findings in the gRNA1 cells which showed no significant change in the mRNA expression of E-cad and VIM and had non-significant change in levels of TGFβ1 expression.

Consistent with the observation of EMT or EMT-like phenomenon, T2-KD cells and gRNA2 cell line in which TIMP-2 expression was suppressed, all demonstrated enhanced invasion in Matrigel compared to their control and parental counterparts. In addition, increased sensitivity to PTX was only observed in the gRNA2 cells; interestingly, gRNA1 cells showed an increase in resistance to the PTX chemotherapy. Previous studies have shown that EMT-transformed cancer cells or particularly TWIST-mediated EMT transformed breast cancer cells are more resistant to therapy treatment due to enrichment of cancer-stem like cells [54]. Studies from our and other laboratories have shown that activation of STAT3 in response to chemotherapy treatment as pre-requisite for cancer stemness and related therapy-resistance in ovarian cancer cells [25, 26, 55–59]. We have previously shown, that unlike the vector control cells, siRNA knockdown of TIMP-2 in OVCAR4 ovarian cancer cell line rendered the transfected cells sensitive to PTX treatment. This resulted in failure of the TIMP-2 knocked down in OVCAR4 cells to activate STAT3 pathway and concurrent enhancement of the expression of TIMP-2 as observed in the control cell line [24]. These observations suggest a link between concurrent enhancement in TIMP-2 expression and activation of STAT3 in PTX resistant cells. In that context, activation of STAT3 has been shown to be critical in regulating EMT and other tumorigenic functions in cancer [55, 60–62]. We and others have previously shown constitutively active STAT3 to be involved with ovarian tumorigenesis and chemotherapy resistance [25, 63–65]. An association between activated STAT3 with integrin b6 promoter, another marker of epithelial ovarian cancer progression [66, 67], has been shown to promote tumorigenesis in prostate cancer [68]. Whether such associations between TIMP-2 and activated STAT3 exists are yet to be determined.

An alternative hypothesis for the difference in sensitivity to PTX in gRNA1 and gRNA2 cells may be due to the differences in the mRNA expression of cell cycle regulators CDC25B and CDC25C in the respective cells. Both CDC25B and CDC25C promote mitosis but have distinct roles in the regulation of the mitotic process in cells [69]. Overexpression of CDC25B rapidly causes the cells in S and M2-phases of the cell cycle to enter mitosis irrespective of DNA replication [69]. However, CDC25C overexpression in cells is much less efficient than CDC25B overexpression in pushing cells in M2 phase to enter mitosis. As PTX induces cell cycle (G2/M) mitotic arrest by destabilizing the microtubule dynamics of cells, upregulation of CDC25B in gRNA1 cells may provide greater advantage to overcome the toxic anti-mitotic effect of PTX. However, concurrent upregulation of both CDC25B and CDC25C in gRNA2 clones may not be as efficient in overcoming the anti-mitotic arrest induced by PTX as only upregulation of CDC25B in gRNA1 [69].

In addition, production of reactive oxygen species (ROS) (oxidative stress) and initiation of stress-activated pathways in response to PTX treatment may also differ in both cell types, which could also determine sensitivity to PTX [70]. It is well documented that chemotherapy-induced ROS and oxidative stress can regulate STAT3 transcriptional activity in ovarian cancer cells [71–73], creating another layer of complexity in the comparative responses of gRNA1 and gRNA2 cells to PTX.

We also report that the morphology of floating spheroids of parental, CRISPR control, gRNA1 and gRNA2 cells varied when grown in 3D culture. While the parental, CRISPR control and gRNA1 cells formed very elongated sheet-like floating cell aggregates, gRNA2 cells formed round compact spheroids, implying that the biology of gRNA1 and gRNA2 cells varied significantly when cultured as 3D structures. Consistent with compact spheroid structure, Day 8 spheroids derived from gRNA2 cells had a significantly high mRNA expression of MMP-2, MMP-14, E-Cad and N-Cad expression compared to spheroids from gRNA1 clones or control OVCAR5 cells. In that context, we have shown enhanced secretion of MMP-2 and MMP-9 in ovarian cancer cells cultured as spheroids compared to monolayer cultures [74].

Spheroids from all cells reattached onto tissue culture flasks as monolayer cultures within 10 h of seeding. Significantly high proliferation in 24-h reattached gRNA1 cells, deduced by high Ki67 staining and significantly higher mRNA expression of KRT14, is consistent with the in vivo results where gRNA1 cells injected into mice produced larger ascites volumes than mice injected with either control or parental cells and had more extensive tumour burden and tumour infiltration in peritoneal organs (liver and pancreas) than tumours derived from either control or parental cell lines. These studies are also consistent with previous studies which showed KRT14 enriched ‘leader cells’ to reside in the periphery of ovarian cancer spheroids and to be directly involved with ECM invasion and degradation [32, 75]. In contrast, none of the gRNA2 cells xenotransplanted in mice produced ascites, revealed no tumour infiltration of the organs tested and survived significantly longer than gRNA1, control and parental cells. These in vivo results suggests that the tumorigenic phenotype in ovarian cancer cells may vary depending on the level of expression of TIMP-2; it can be provoked and aggravated by a pronounced deficiency of TIMP-2 expression, as shown in gRNA1 cells. Based on these findings it can be postulated that an array of different pathways controlling tumorigenesis can be activated according to the level of expression of TIMP-2 in ovarian cancer cells.

The above results indicate that 3D cell line model correlated with the xenograft model and is different in relation to phenotypic output of cells observed in 2D monolayer cultures. This is consistent with the literature where differences in the phenotype of the same cells have been portrayed when grown in 2D vs 3D cultures [76]. This may result due to differences in the gene expression induced by the architectural, ECM and cytoskeletal rearrangement in the cells grown in 2D vs 3D cultures enabling the transcription of different sets of genes and has been described previously in monolayers vs spheroids cultures [76].

The TIMP: MMP balance has been shown to contribute to tumour progression [77–79]. Recently, an unbalanced expression of TIMP: MMP genes in tumours was correlated with an aberrant epigenotype in various gene promoters [80]. Correction of these malignant epigenotypes by nuclear modelling was shown to rebalance the tumorigenic gene expression profile resulting in altered tumour cell morphology, attenuation of migration and invasion in vitro and reduced tumour growth in vivo [80]. In that context, the cytotoxic effect of chemotherapy has been shown to induce an imbalance in TIMP: MMP which if not corrected results in tumour progression and recurrence. An imbalance between the serum TIMP-2 and MMP9 was shown to predict disease progression in patients with metastatic renal cell carcinoma in response to sunitinib treatment [81]. Even though there was no significant difference in the serum levels of MMP-9 and TIMP-2 at diagnosis, the TIMP-2: MMP9 ratio was significantly higher at the time of progression in non-responders versus responders in response to sunitinib treatment in metastatic renal cell carcinoma [81]. As TIMP-2 has been reported to be a major regulator of matrisome biology [34], gaining an understanding on how different degrees of alterations in TIMP-2 expression in ovarian cancer impacts on different MMPs and their levels in cancer cells and how it may affect cancer cell chemosensitivity, migration/invasion and tumorigenic growth potentially poses a major advancement not only in understanding of TIMP-2 mediated ovarian tumour biology but may also lay the foundation of TIMP-2-based targeted therapy.

In conclusion, the findings described demonstrate a complex role of TIMP-2 in ovarian cancer progression. Despite indicating several limitations of the CRISPR/Cas9 methodology, this technology identified two different cell lines displaying different degrees of TIMP-2 protein suppression. The resultant gRNA1 and gRNA2 cells displayed a degree of molecular and functional dissimilarities in in vitro assays but substantial molecular and functional differences in long-term 3D cultures and in an in vivo mouse model. Although a balance of TIMPs and MMPs has been described to be critical for ECM proteolysis regulating cancer progression [34], it should be emphasized that the composition of the ECM is also regulated by inflammatory and other biological signals received within the tumour microenvironment. As initiation of inflammation and its sustained level is a major contributor of ovarian tumorigenesis [82, 83], it can be postulated that the differences in the ratios of TIMP-2 and MMPs may be a tipping point in controlling the tumorigenic behaviour of ovarian cancer cells. Ovarian cancer cells with less availability of TIMP-2 and more of MMPs as in the case of gRNA1 cells, may generate aggressive tumours with a greater degree of tumour burden and metastasis resulting in infiltration to the peritoneal organs. On the other hand, when there is relatively more TIMP-2 and consequently less active MMPs, as in the case of gRNA2 cells, there is less autocrine availability of active MMPs, the generated tumour burden is smaller, and tumours are less aggressive, contained, and non-infiltrating but inflammatory in phenotype. As a result, tumours with a high TIMP-2/MMP ratio may be more sensitive to chemotherapy due to their docile non-aggressive nature in contrast to tumours with a low TIMP-2/MMP ratio which, because of their inherent proteolytic nature, may be more resistant to chemotherapy.

**Fig. 14:**
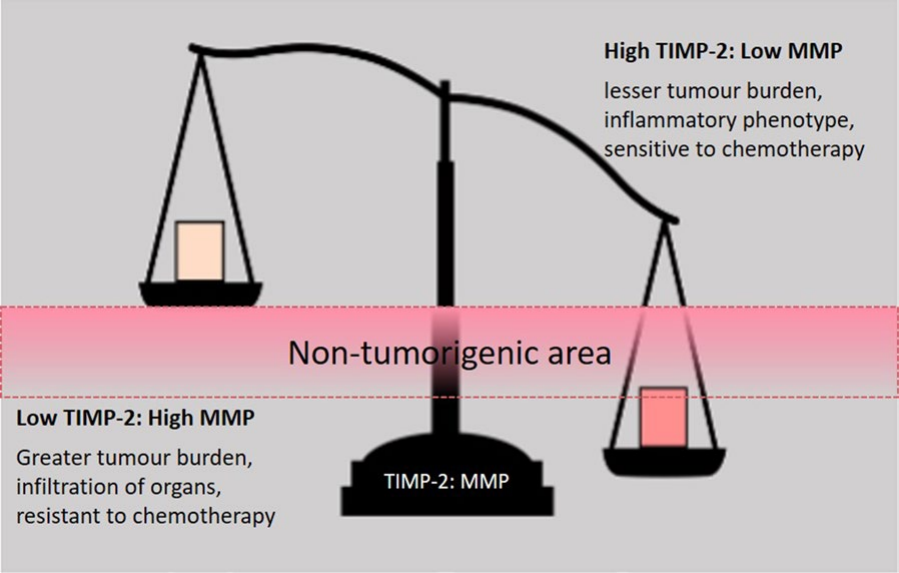
Proposed model of TIMP-2/MMP ratio that controls ovarian tumorigenesis and chemotherapy (PTX) sensitivity. The model proposes that the ratio of TIMP-2/MMPs may decide the fate of ovarian cancer cells. Ovarian cancer cells with a low TIMP-2/MMP ratio (less availability of TIMP-2 and more MMPs) as in the case of the gRNA1 cells, may generate aggressive EMT-induced tumours with a greater degree of tumour burden and metastasis. These aggressive tumour cells due to their inherent proteolytic nature remodel tumour ECM to facilitate metastasis and are naturally resistant to chemotherapy. On the other hand, when the TIMP-2/MMP ratio is higher (more availability of TIMP-2) as seen in the case of gRNA2 cells, TGFβ induced EMT is displayed, but the tumours are less aggressive, inflammatory but non-infiltrating and sensitive to chemotherapy

A model of disruption in TIMP-2 expression and its consequences in ovarian tumorigenesis has been described in Fig. 14.

## Supplementary Information

**Additional file 1: Figure S1.** siRNA suppression of TIMP-2 in the OVCAR5 cell line. Diagram showing the location of single siRNA duplexes A, B, C in the TIMP-2 gene. **Figure S2.** CRISPR/Cas9 editing of the TIMP-2 gene in the OVCAR5 cell line. **A** Diagram showing the configuration of TIMP-2 CRISPR/Cas9 plasmid. The TIMP-2 linear donor plasmid is ~ 2.74 kb and incorporates the donor GFP and puromycin (under the EF1a promoter). The transfection involves integration of Cas9/gRNA and the linear donor plasmid (GFP and puromycin) genes into cells. The Cas9 targets the TIMP-2 gene in exon1, guided by two gRNA sequences [the gRNA1 (yellow) and gRNA2 (pink)]. The donor genes can be inserted in the cells by transcription in the forward or reverse directions. In both situations, interruption of TIMP-2 expression coinciding with puromycin resistance and GFP expression should occur. (Figure adapted from https://www.origene.com/catalog/gene-expression/knockout-kits-crispr/kn409796/timp2-human-gene-knockout-kit-crispr). **B** Puromycin “death” curve in the OVCAR5 cell line. The OVCAR5 cell line was incubated with puromycin concentrations ranging from 0 to 320 µg/mL followed by an MTT assay of OVCAR5 cells after 48 h. The concentration of 3 µg/mL (indicated by red arrow) was used for puromycin selection. Values are mean ± SEM and graph is representative of three experiments done in triplicate. **C** CRISPR transfected OVCAR5 cells after puromycin selection and GFP sorting. OVCAR5 cells were transfected with CRISPR/Cas9 vectors plus the donor plasmid containing puromycin and GFP genes. After puromycin selection, cells were GFP sorted and visualized under a confocal microscope. After a second GFP sorting, GFP fluorescence was only seen in gRNA2 cells but not in gRNA1 cells. 20× magnification; scale bar 1000 µM. **D** MTT assay of OVCAR5 TIMP-2 CRISPR/Cas9 transfected and Control cells. After 48 h of incubation the concentration of puromycin that killed 50% of cells was determined (IC50 values). Values are mean ± SEM and the graph is representative of three experiments done in triplicate. **Figure S3.** Expression of cellular TIMP-2 and corresponding GAPDH by Western blot. Representative full image of a Western blot of TIMP-2 and GAPDH proteins on the cell lysates of parental, CRISPR/Cas9 treated control, gRNA1 and gRNA2 cell lines. **Figure S4.** Quantification of EdU stained OVCAR5 parental, CRISPR control, and TIMP-2 knocked down gRNA2 and gRNA1 cells. The cells were stained with EdU and propidium iodide (PI) as described in “Methods”. **A** Flow cytometer representation of percentage of EdU stained cells in S-phase of the cycle for CRISPR transfected cell lines. **B** Flow cytometer representation of negative controls used to calculate the percentage of EdU stained cells in S-phase of the cycle for the OVCAR5 parental cell line. Red rectangles indicate the areas analysed for EdU positive cells. **Table S1.** Primers used in the study.

## Data Availability

The data presented in the study are not publicly available as it forms part of a PhD dissertation in progress. However, the data can be made available to a corresponding author on reasonable request.

## Abbreviations

TIMP: Tissue inhibitor of metalloproteinases
T2-KD: SiRNA mediated TIMP-2 knocked down cells
siRNA: Small interfering RNA
CRISPR: Clustered regularly interspaced short palindromic repeat DNA sequences
Cas9: Caspase 9
gRNA1: CRISPR/Cas9 guide RNA1 mediated TIMP-2 knocked down cell line
gRNA2: CRISPR/Cas9 guide RNA2 mediated TIMP-2 knocked down cell line
TME: Tumour microenvironment
Cont: Scrambled vector transfected control cells
P: Parental cells treated only with transfection reagent
Unt: Untreated cells
MMP: Matrix metalloproteinase
EMT: Epithelial mesenchymal transition
CSCs: Cancer stem cells
STAT3: Signal transducer and activator of transcription 3
E-Cad: E-cadherin
N-Cad: N-cadherin
VIM: Vimentin
CA125: Cancer antigen125
MTT: 3-(4,5-Dimethylthiazol-2-yl)-2,5-diphenyltetrazolium bromide
EdU: 5-Ethynyl-2′-deoxyuridine
SDS-PAGE: Sodium dodecyl sulfate-polyacrylamide gel electrophoresis
DAPI: 4′,6-Diamidino-2-phenylindole
qRT-PCR: Real-Time Quantitative Reverse Transcription Polymerase Chain Reaction
PI: Propidium iodide
IP: Intraperitoneal
