# Supplementary material for: Knock down of TIMP-2 by siRNA and CRISPR/Cas9 mediates diverse cellular reprogramming of metastasis and chemosensitivity in ovarian cancer
Source: Cancer Cell Int. 2022 Dec 30;22:422. doi: 10.1186/s12935-022-02838-x (PMC9805260; doi:10.1186/s12935-022-02838-x)
Supplement: Supplementary file 1 — Additional file 1: Figure S1. siRNA suppression of TIMP-2 in the OVCAR5 cell line. Diagram showing the location of single siRNA duplexes A, B, C in the TIMP-2 gene. Figure S2. CRISPR/Cas9 editing of the TIMP-2 gene in the OVCAR5 cell line. A Diagram showing the configuration of TIMP-2 CRISPR/Cas9 plasmid. The TIMP-2 linear donor plasmid is ~ 2.74 kb and incorporates the donor GFP and puromycin (under the EF1a promoter). The transfection involves integration of Cas9/gRNA and the linear donor plasmid (GFP and puromycin) genes into cells. The Cas9 targets the TIMP-2 gene in exon1, guided by two gRNA sequences [the gRNA1 (yellow) and gRNA2 (pink)]. The donor genes can be inserted in the cells by transcription in the forward or reverse directions. In both situations, interruption of TIMP-2 expression coinciding with puromycin resistance and GFP expression should occur. (Figure adapted from https://www.origene.com/catalog/gene-expression/knockout-kits-crispr/kn409796/timp2-human-gene-knockout-kit-crispr). B Puromycin “death” curve in the OVCAR5 cell line. The OVCAR5 cell line was incubated with puromycin concentrations ranging from 0 to 320 µg/mL followed by an MTT assay of OVCAR5 cells after 48 h. The concentration of 3 µg/mL (indicated by red arrow) was used for puromycin selection. Values are mean ± SEM and graph is representative of three experiments done in triplicate. C CRISPR transfected OVCAR5 cells after puromycin selection and GFP sorting. OVCAR5 cells were transfected with CRISPR/Cas9 vectors plus the donor plasmid containing puromycin and GFP genes. After puromycin selection, cells were GFP sorted and visualized under a confocal microscope. After a second GFP sorting, GFP fluorescence was only seen in gRNA2 cells but not in gRNA1 cells. 20× magnification; scale bar 1000 µM. D MTT assay of OVCAR5 TIMP-2 CRISPR/Cas9 transfected and Control cells. After 48 h of incubation the concentration of puromycin that killed 50% of cells was determined (IC50 values). Values [file 12935_2022_2838_MOESM1_ESM.docx]

**Additional file 1**

**
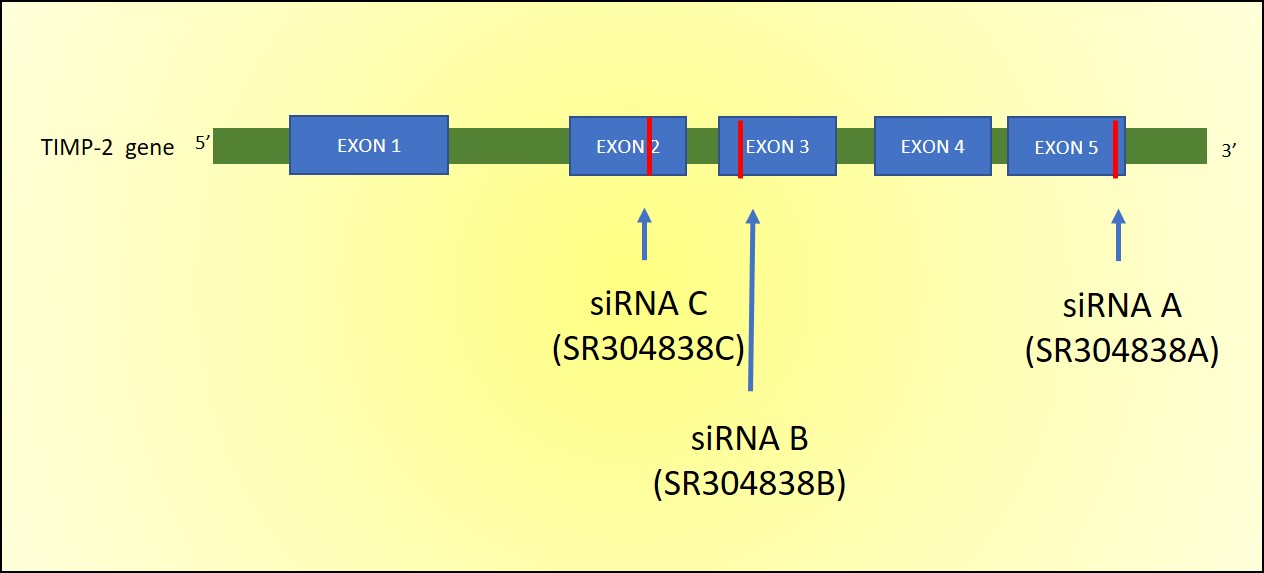
**

**Figure S1:** siRNA suppression of TIMP-2 in the OVCAR5 cell line. Diagram showing the location of single siRNA duplexes A, B, C in the TIMP-2 gene.


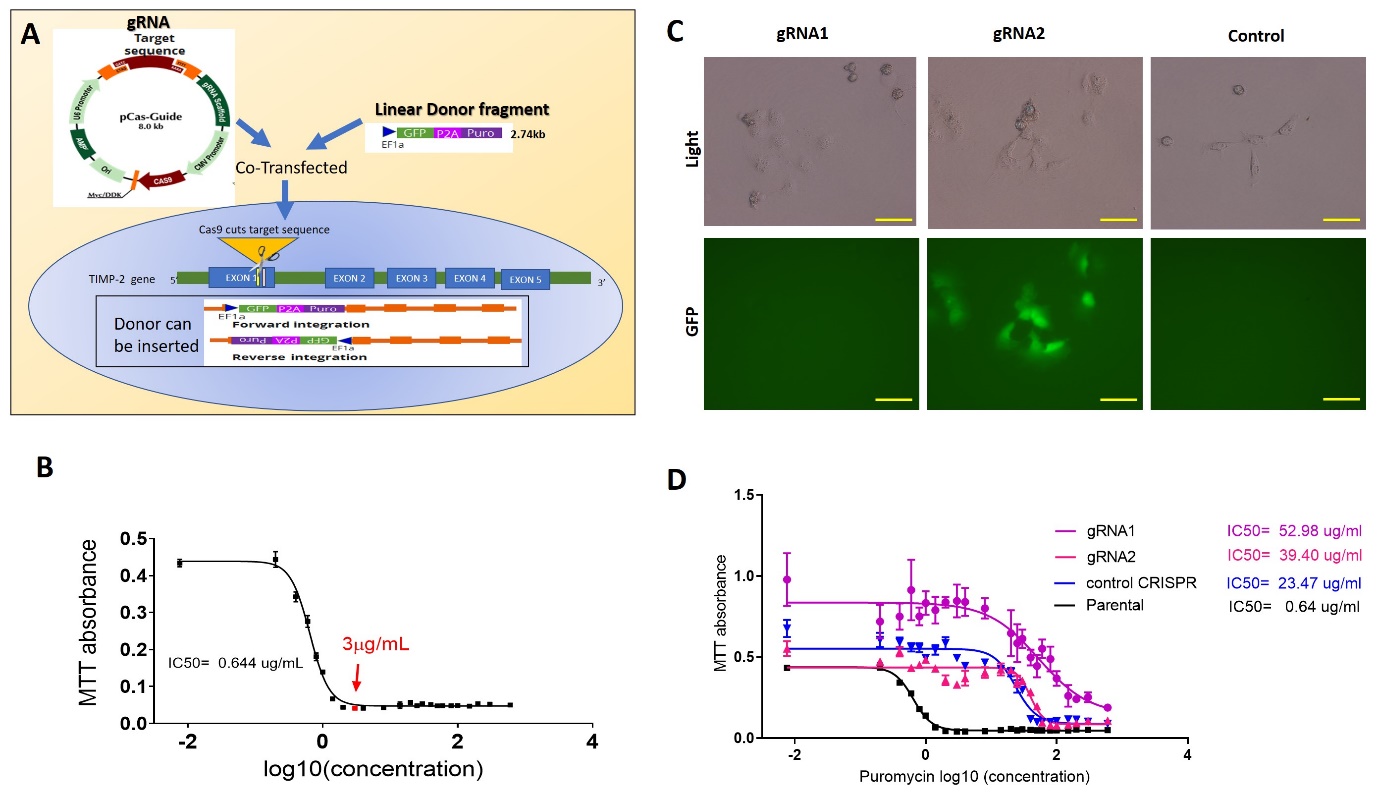


**Figure S2:** CRISPR/Cas9 editing of the TIMP-2 gene in the OVCAR5 cell line. (A) Diagram showing the configuration of TIMP-2 CRISPR/Cas9 plasmid. The TIMP-2 linear donor plasmid is ~2.74kb and incorporates the donor GFP and puromycin (under the EF1a promoter). The transfection involves integration of Cas9/gRNA and the linear donor plasmid (GFP and puromycin) genes into cells. The Cas9 targets the TIMP-2 gene in exon1, guided by two gRNA sequences [the gRNA1 (yellow) and gRNA2 (pink)]. The donor genes can be inserted in the cells by transcription in the forward or reverse directions. In both situations, interruption of TIMP-2 expression coinciding with puromycin resistance and GFP expression should occur. (Figure adapted from https://www.origene.com/catalog/gene-expression/knockout-kits-crispr/kn409796/timp2-human-gene-knockout-kit-crispr). (B) Puromycin “death” curve in the OVCAR5 cell line. The OVCAR5 cell line was incubated with puromycin concentrations ranging from 0-320µg/mL followed by an MTT assay of OVCAR5 cells after 48 hours. The concentration of 3µg/mL (indicated by red arrow) was used for puromycin selection. Values are mean + SEM and graph is representative of three experiments done in triplicate. (C) CRISPR transfected OVCAR5 cells after puromycin selection and GFP sorting. OVCAR5 cells were transfected with CRISPR/Cas9 vectors plus the donor plasmid containing puromycin and GFP genes. After puromycin selection, cells were GFP sorted and visualized under a confocal microscope. After a second GFP sorting, GFP fluorescence was only seen in gRNA2 cells but not in gRNA1 cells. 20X magnification; scale bar 1000µM. (D) MTT assay of OVCAR5 TIMP-2 CRISPR/Cas9 transfected and Control cells. After 48 hours of incubation the concentration of puromycin that killed 50% of cells was determined (IC50 values). Values are mean + SEM and the graph is representative of three experiments done in triplicate.


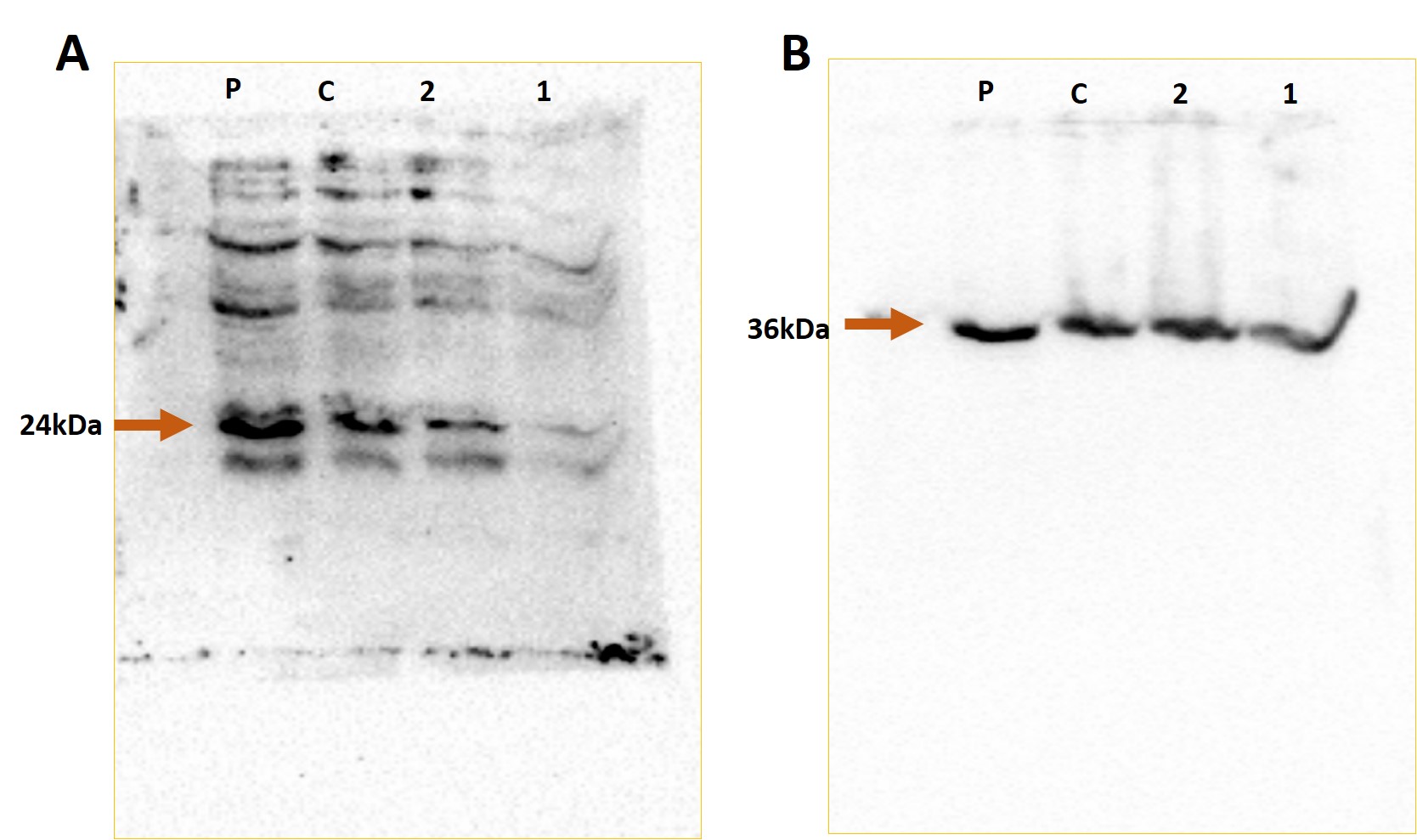


**Figure S3:**  Expression of cellular TIMP-2 and corresponding GAPDH by Western blot. Representative full image of a Western blot of TIMP-2 and GAPDH proteins on the cell lysates of parental, CRISPR/Cas9 treated control, gRNA1 and gRNA2 cell lines.


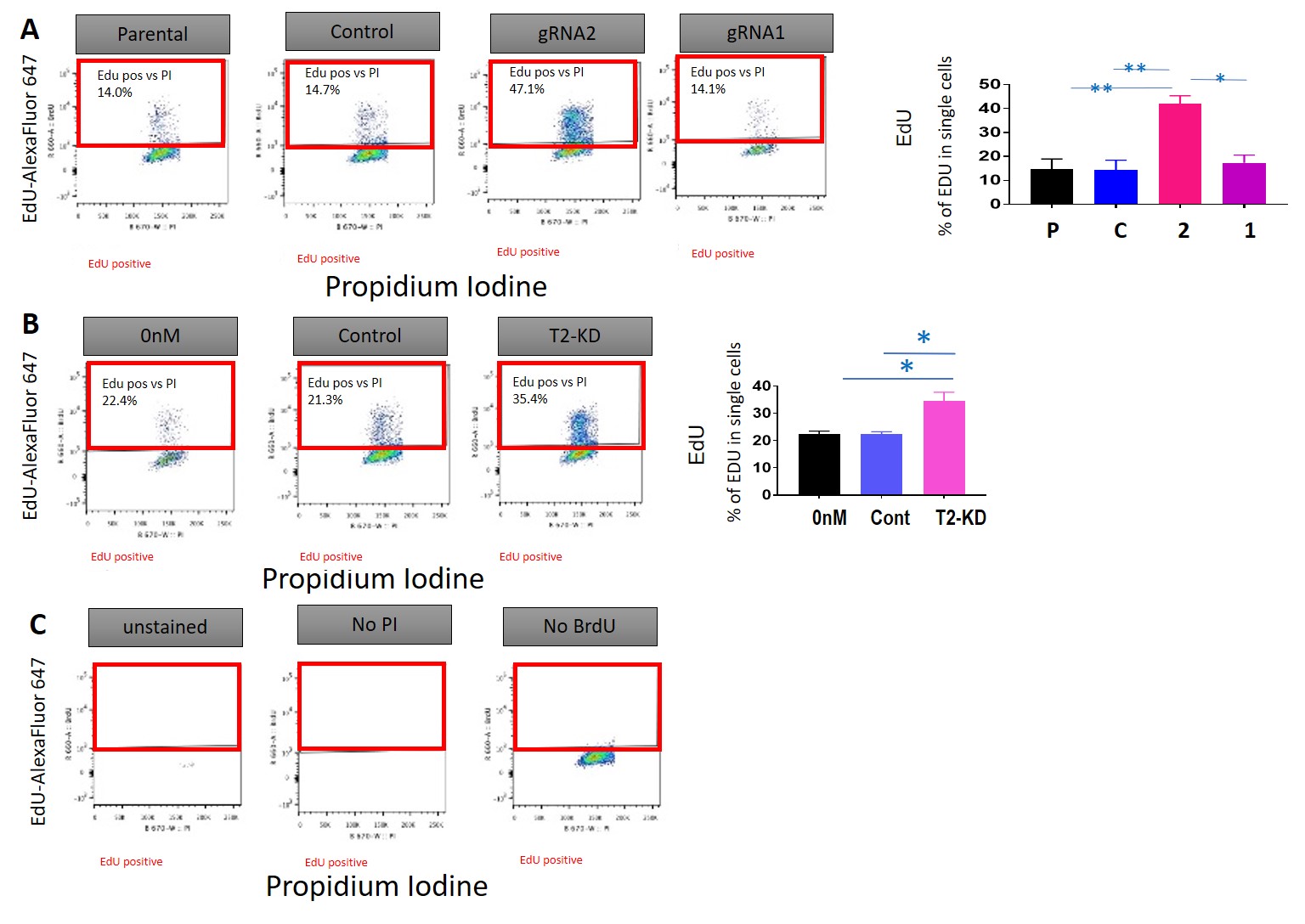


**Figure S4:** Quantification of EdU stained OVCAR5 parental, CRISPR control, and TIMP-2 knocked down gRNA2 and gRNA1 cells. The cells were stained with EdU and propidium iodide (PI) as described in Methods. (A) Flow cytometer representation of percentage of EdU stained cells in S-phase of the cycle for CRISPR transfected cell lines. (B) Flow cytometer representation of negative controls used to calculate the percentage of EdU stained cells in S-phase of the cycle for the OVCAR5 parental cell line. Red rectangles indicate the areas analysed for EdU positive cells.

**Table S1. Primers used in the study.**

| Gene symbol | Sequences (5' - 3') | Accession # | Product size (bp) | Fluorescence Capture (°C) |
| --- | --- | --- | --- | --- |
| 18S | F GTAACCCGTTGAACCCCATT  R CCATCCAATCGGTAGTAGCG | NR_003286.1 | 153 | 78 |
| CDC25A | F CTACTCATCCCTGCCCTCTG  R GTCCTCTCCCCCACATTTTT | NM_001789 | 217 | 80 |
| CDC25B | F CCTCCGCTCAAAATCACTGT  R CCACGATGTTGCTGAACTTG | NM_021873.3 | 188 | 81 |
| CDC25C | F AGTGGGCAAATTTCTTGGTG  R TCCCAGCTAAATGCACTTCC | NM_001790.4 | 188 | 81 |
| KRT14 | F TTCTGAACGAGATGCGTGAC  R GCAGCTCAATCTCCAGGTTC | NM_000526.5 | 189 | 79-81 |
| mKi67 | F TTGGTACTGGGGGAGGGAGA  R TGGGAGGCGAAAAAGTAAAA | NM_002417.4 | 188 | 78 |
| MMP-2 | F TTGACGGTAAGGACGGACTC  R ACTTGCAGTACTCCCCATCG | NM_004530.4 | 153 | 80-83 |
| MT1-MMP/MMP-14 | F GCTCCGAGGGGAGATGTTTG  R CAGCTCCTTAATGTGCTTGGG | NM_004995.2 | 235 | 83 |
| N-CAD/CDH2 | F AAACAGCAACGACGGGTTAG  R CTTAGGATTGGGGGCAAAAT | NM_001792.3 | 195 | 78 |
| SLUG/SNAI2 | F TTCGGACCCACACATTACCT  R GCAGTGAGGGCAAGAAAAAG | NM_003068 | 122 | 79-81 |
| SNAIL | F GGTTCTTCTGCGCTACTGCT  R TAGGGCTGCTGGAAGGTAAA | NM_005985 | 157 | 83 |
| TGeta1-Ligand | F CAACAATTCCTGGCGATACCT  R GCTAAGGCGAAAGCCCTCAAT | NM_000660 | 136 | 79-82 |
| TIMP-1 | F TGACATCCGGTTCGTCTACA  R GTTTGCAGGGGATGGATAAA | NM_003254.2 | 248 | 85 |
| TIMP-2 | F CCGCAACAGGCGTTTTGCAA  R TCACTTCTCTTGATGCAGGC | NM_003255.4 | 494 | 85 |
| TIMP-3 | F TTCTGCAACTCCGACATCGT  R ATGCAGGCGTAGTGTTTGGA | NM_000362.4 | 452 | 83 |
| TWIST1 | F GTCCGCAGTCTTACGAGGAG  R CCAGCTTGAGGGTCTGAATC | NM_000474.3 | 159 | 88 |
| VIM | F CCTACAGGAAGCTGCTGGAA  R GGTCATCGTGATGCTGAGAA | NM_003380.3 | 198 | 75 |
